# Next-Generation Sequencing in Colorectal Cancer: Real-World Molecular Profiling and Clinical Correlations

**DOI:** 10.3390/ijms27156930

**Published:** 2026-08-01

**Authors:** Afonso Cunha, Carolina Robalo, Carolina Lemos, Nuno Jorge Lamas, Marisa Domingues dos Santos

**Affiliations:** 1 School of Medicine and Biomedical Sciences, University of Porto, Rua Jorge de Viterbo Ferreira, 228, 4050-313 Porto, Portugal; 2 Unidade de Cirurgia Colorretal, Serviço de Cirurgia Digestiva e Extradigestiva, Unidade Local de Saúde de Santo António, 4050-313 Porto, Portugal; 3 Unit for Multidisciplinary Research in Biomedicine, School of Medicine and Biomedical Sciences, University of Porto, Rua Jorge de Viterbo Ferreira, 228, 4050-313 Porto, Portugal; 4 ITR—Laboratory for Integrative and Translational Research in Population Health, 4050-313 Porto, Portugal; 5 Serviço de Anatomia Patológica, Clínica de Patologia e Genética Unidade Local de Saúde de Santo António, 4050-313 Porto, Portugal; 6 Escola de Medicina, Universidade do Minho, Campus de Gualtar, Edifício 19, 4710-057 Braga, Portugal

**Keywords:** colorectal cancer, next-generation sequencing, molecular profiling, somatic alterations, clinical phenotypes

## Abstract

Next-generation sequencing (NGS) has become a cornerstone of precision oncology in colorectal cancer (CRC), although its role in routine patient stratification remains incompletely defined. This retrospective single-centre study characterised the molecular landscape of clinically selected CRC patients undergoing routine NGS and explored associations between genomic alterations and clinicopathological features. 97 eligible patients who underwent targeted NGS using two validated sequencing platforms were selected. Demographic, clinicopathological, and molecular data were integrated, and associations were evaluated using descriptive statistics, exploratory association testing, principal component analysis, and multiple correspondence analysis. At least one reportable genetic alteration was identified in 91 patients (93.8%), comprising 198 alteration events across multiple cancer-related genes. The most frequently altered genes with pathogenic or likely pathogenic variants were *TP53* (55.7%), *KRAS* (40.2%), *PIK3CA* (18.6%), and *BRAF* (11.5%), while microsatellite instability was detected in 19.1% of evaluable tumours. Exploratory analyses identified associations between selected molecular alterations and clinicopathological characteristics. However, these were generally modest, frequently limited by small subgroup sizes, and none survived Benjamini–Hochberg correction. Principal component analysis demonstrated substantial molecular heterogeneity without defining distinct clinicopathological subgroups. Routine targeted NGS provides detailed molecular characterisation of CRC and generates information relevant to biomarker-informed clinical decision-making in real-world practice. However, targeted panels alone were insufficient to establish robust molecular subgroups in this retrospective cohort. These findings highlight the biological complexity of CRC and support larger prospective studies incorporating broader molecular profiling to improve precision patient stratification and optimise personalised therapeutic strategies.

## 1. Introduction

Colorectal cancer (CRC) remains a major global health burden, ranking as the third most frequently diagnosed cancer and the second leading cause of cancer-related death worldwide [1,2,3,4]. In 2022, nearly 2 million new cases were estimated globally. In Portugal, CRC also represents a major public health challenge, with 10,575 new cases estimated in 2022, making it the most incident cancer overall and a leading cause of cancer-related death [2].

CRC is a multifactorial disease resulting from the interplay between environmental exposures, lifestyle determinants, host factors, and inherited susceptibility [1,3,5,6]. Established modifiable risk factors include consumption of red and processed meat, diet low in fruits and vegetables, obesity, metabolic syndrome, diabetes, alcohol consumption, and tobacco exposure, whereas physical activity and healthier dietary patterns, including a diet high in natural fibre or whole grains, appear protective [3,7].

Although CRC remains a disease predominantly of older adults, early-onset CRC, usually defined as diagnosis before the age of 50 years, has emerged as a relevant entity due to its increasing incidence in several populations [5]. Early-onset CRC is clinically and biologically heterogeneous, with reported differences in tumour location, stage at presentation, MSI/MMR status and selected driver alterations across studies [8,9,10,11].

At the molecular level, CRC develops through a multistep process driven by the progressive accumulation of genetic and epigenetic alterations [12,13,14]. Colorectal carcinogenesis occurs through partially overlapping molecular pathways. This leads to biological tumour heterogeneity, which has major implications for prognosis, therapeutic stratification, and the growing transition from conventional histopathological classification to molecularly informed management [5].

The classical adenoma–carcinoma sequence is commonly associated with chromosomal instability and stepwise accumulation of alterations involving *APC*/*WNT*/*β-catenin* signalling, *RAS*/*RAF*/*MAPK* activation, *PI3K*/*AKT*/*mTOR* signalling, *TGF-β*/*SMAD* disruption and TP53/cell-cycle dysregulation [12,13,15,16,17]. Other tumours arise through microsatellite instability due to deficient mismatch repair, either sporadically or in the context of Lynch syndrome [13,16,17]. A further route is the serrated/CpG island methylator phenotype pathway, often associated with *BRAF* or *KRAS* mutations, epigenetic dysregulation and, in some cases, *MLH1* methylation and *MSI* [16,18,19,20]. These routes help explain the biological heterogeneity of CRC and provide the rationale for analysing alteration-based molecular profiles in relation to clinical presentation features [12,13,17,21].

Molecular characterization is now central to clinical decision-making [22,23,24,25]. Universal assessment of mismatch repair deficiency or microsatellite instability is recommended in CRC and is particularly relevant during the initial work-up of rectal tumours [22,23]. In metastatic disease, evaluation of *RAS* and *BRAF* alterations is mandatory to guide anti-*EGFR*-based strategies, and additional biomarkers such as *HER2* amplification may identify candidates for targeted treatment [24]. As the number of clinically relevant biomarkers increases, and given that phenotypic features can be overlapping or ambiguous, the limitations of sequential single-gene testing have become increasingly evident [26].

Next-generation sequencing (NGS) has emerged as a key tool in precision oncology by enabling the simultaneous assessment of multiple genomic alterations from a single specimen [27]. In CRC, this approach can identify actionable and biologically informative alterations, including point mutations, small insertions/deletions, selected copy-number changes and gene fusions, thereby supporting molecular profiling in appropriate clinical settings [28]. Larger NGS panels, besides analysing a larger array of genes, also permit the evaluation of relevant metrics like tumour mutational burden (TMB), microsatellite instability (MSI), and homologous recombination deficiency (HRD).

Thus, beyond the identification of currently actionable genomic alterations in CRC, tumour-based NGS may also contribute to the identification of other potential actionable or prognostically relevant molecular alterations. Several studies have shown that somatic mutational profiles differ according to the age at diagnosis, tumour location and precursor-lesion pathway, supporting the concept that CRC comprises biologically distinct clinicomolecular subgroups rather than a single homogeneous entity [8,21,29,30,31,32].

The growing understanding of colorectal carcinogenesis has highlighted the existence of patient subgroups whose clinical presentation, disease evolution, or underlying predisposition differs from that observed in conventional sporadic CRC. These include patients with early-onset CRC, synchronous or metachronous tumours, significant polyposis phenotypes, hereditary cancer syndromes such as Lynch syndrome and familial adenomatous polyposis, and individuals with a strong personal or family history suggestive of inherited susceptibility. Such clinical phenotypes are likely to reflect distinct biological mechanisms of tumour development and potentially require different approaches to screening, surveillance, diagnosis, treatment, and prognostic assessment [30,32,33,34,35,36,37].

In this context, integrating tumour genomic profiles with clinical, pathological, and endoscopic features may help identify clinicomolecular patterns associated with these distinct clinical phenotypes [30,32,33]. Although such findings alone are insufficient to establish tailored management strategies, they may provide valuable insights into CRC heterogeneity and contribute to the future development of more individualized approaches to prevention, surveillance, and treatment [33,34].

Beyond immediate therapeutic actionability, tumour molecular profiling may provide biological and prognostic information. In metastatic CRC, alterations involving *SMAD4* and *FBXW7* have been associated with poorer outcomes, particularly in *TP53*-mutated tumours, and molecular subtypes reflecting distinct carcinogenesis pathways have been linked to differences in patient survival [29,38,39,40]. Therefore, even when no directly actionable alteration is identified, genomic profiling of colorectal tumours and selected precursor lesions may contribute to genotype–phenotype correlation and prognostic stratification, although its clinical implications depend on tumour context, assay scope, and integration with clinicopathological data [29,30,31,32,38,39,40].

While molecular profiling in CRC plays an expanding role, current evidence primarily focuses on advanced disease, therapeutic actionability, broad genomic landscapes, prognostic signatures, or predefined molecular and clinicopathological subgroups [8,28,29,31,32,33]. Less is known about whether the somatic genetic alterations detected by routine targeted NGS can distinguish clinically defined CRC phenotypes that differ from conventional sporadic disease presentations. In particular, it remains unclear whether patients presenting with early-onset disease, multiple primary tumours, polyposis phenotypes, or personal and familial features suggestive of inherited susceptibility exhibit distinct somatic genetic patterns detectable by the targeted-NGS panels currently used in clinical practice.

The clinical value of NGS extends beyond identifying hereditary syndromes or actionable mutations, supporting a precision-surgery approach in which molecular biomarkers complement conventional clinicopathological risk stratification.

Therefore, the primary objective of this study was to characterize the molecular alterations identified by routine targeted NGS in colorectal tumours and determine whether clinically defined CRC phenotypes exhibit distinct somatic genetic profiles. Secondary objectives were to explore clinicopathological associations and identify patient-level molecular patterns that may support precision oncology and future precision surgical strategies.

## 2. Results

### 2.1. Study Population and Baseline Characteristics

The final analytical cohort included 97 patients with CRC and available NGS data. The mean follow-up was 17.36 ± 13.6 months. The cohort included 45 women (46.4%) and 52 men (53.6%). At last follow-up, 48 patients (49.5%) were alive without evidence of disease, 39 (40.2%) were alive with disease, and 10 (10.3%) had died from the disease.

Most patients had a colonic primary tumour (73/97, 75.3%), whereas 24/97 (24.7%) had rectal disease. Four patients (4.1%) had significant polyposis (≥10 colorectal polyps), and no colonoscopy was documented in 22 (22.7%). General cohort characteristics are summarised in Table 1.

### 2.2. Clinicopathological Characteristics of Patients with Tumour

Mean age at diagnosis was 63.2 ± 13.1 years. Most primary tumours were colonic (75.3%), and the predominant histological type was adenocarcinoma (92.8%). The most frequent tumour locations were the right colon (35.1%), the sigmoid colon (26.8%), the rectum (19.6%), the left colon (16.5%), the transverse colon (10.3%), and the rectosigmoid junction (3.1%). Microsatellite instability was detected in 19.1% of evaluable patients (18/94).

At diagnosis, 74/96 tumours (77.1%) were T3–T4, nodal involvement was present in 49/96 (51.0%), and metastatic disease was recorded in 20/96 (20.8%). Metastatic involvement at any time during the disease course was documented in 36/96 patients (37.5%), most commonly in the liver (22.9%), lung (14.6%), and peritoneum (10.4%). During follow-up, a new malignant neoplasm distinct from CRC was documented in 8.2% of patients. Detailed clinicopathological, staging, metastatic-site, and treatment data are presented in Table 2.

### 2.3. Family History Data

Family-history information was incomplete in approximately one third of patients and was retained as a separate category rather than assumed to be negative. Any family history of cancer was documented in 43 patients (44.3%), family history of CRC in 17 (17.5%), cancer in a first-degree relative in 29 (29.9%), and a pattern suggestive of hereditary CRC predisposition in 15 (15.5%) (Table 3).

### 2.4. Clinical Presentation and Predefined Clinical Features

Overall, 51/97 patients (52.6%) fulfilled at least one predefined clinical presentation feature. Because these characteristics were not mutually exclusive and likely reflect biologically heterogeneous conditions, the composite variable was retained only for descriptive purposes and was not used as the primary molecular comparator. The inferential analyses used the prespecified individual categories highlighted in Table 4.

The most frequent feature was CRC diagnosis at ≤50 years (32.0%), family history of CRC (17.5%), previous malignant neoplasm (17.5%), and family history suggestive of CRC predisposition (15.5%). Significant polyposis was uncommon (4.1%). The full distribution of predefined clinical phenotypes is shown in Table 4.

### 2.5. Global NGS Landscape

NGS was performed using the Genexus/Oncomine platform in 87 patients (89.7%) and the MiSeq platform in 10 (10.3%). At least one reportable alteration was detected in 91/97 patients (93.8%). After restricting the principal molecular analysis to P/LP sequence-level alterations, 87/97 patients (89.7%) remained alteration-positive. Most patients had one or two P/LP-altered genes and one or two P/LP-affected pathways (Table 5).

### 2.6. Gene and Pathway-Level NGS Findings

Considering all alterations irrespective of classification, reportable alterations most frequently involved *TP53* (54/97, 55.7%), *KRAS* (40/97, 41.2%), *PIK3CA* (19/97, 19.6%), and *BRAF* (11/97, 11.3%), followed by *ERBB2* (5/97, 5.2%), *NRAS*, and *PTEN* (4/97, 4.1% each), with all other genes altered less frequently. 

**Figure 1:**
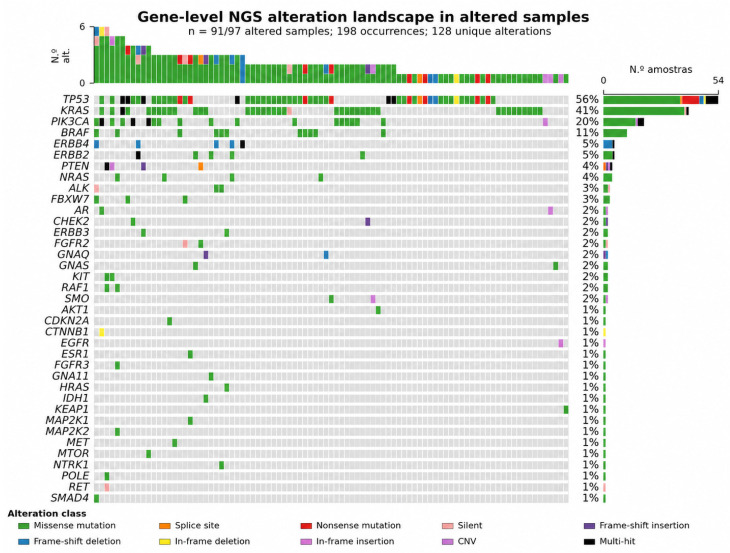
Oncoplot showing all reportable gene-level alterations in samples with at least one NGS alteration (*n* = 91). Genes are ordered by alteration frequency. The upper and right bar plots show the number of alterations per sample and altered samples per gene, respectively. Colours represent alteration classes; Multi-hit indicates more than one reportable alteration in the same gene and sample. Six patients had no reportable alteration and are not displayed. NGS, next-generation sequencing; CNV, copy-number variation.

In the principal P/LP analysis, *TP53* was altered in 54/97 patients (55.7%), *KRAS* in 39/97 (40.2%), *PIK3CA* in 18/97 (18.6%), and *BRAF* in 11/97 (11.3%). Less frequent P/LP alterations involved *NRAS* (4.1%), *PTEN* (3.1%), *ERBB2*, and *ERBB3* (2.1% each), as well as several additional genes (Table 6); gene-level findings for all reportable alterations are provided in Supplementary Table S1. Across the cohort, 198 reportable alteration events (and 128 unique alterations) were catalogued: 159 P/LP sequence-level events, 30 VUS, 5 silent variants, and 4 copy-number variation events. These mutually exclusive analytical categories are detailed in Supplementary Tables S2–S5. Their complete analytical categorisation is presented in Supplementary Table S6. The descriptive oncoplot includes the 91 alteration-positive samples and all reportable events (Figure 1).

Descriptive pathway-level analyses were restricted to pathogenic or likely pathogenic (P/LP) alterations and included the union of genes covered by either NGS platform, using coverage-aware denominators. TP53/cell-cycle regulation and RAS/RAF/MAPK signalling were affected in 57.7% and 54.6% of patients, respectively, followed by PI3K/AKT/mTOR signalling (22.7%) (Table 7). Both shared and platform-specific genes were considered, and genes not covered by the platform used for a given patient were classified as untested rather than unaltered.

The full catalogue of pathogenic and likely pathogenic variants is provided in Supplementary Table S2.

Missense mutations represented 162/198 reportable events (81.8%). The remaining classes included frameshift deletions (5.1%), nonsense mutations (4.5%), silent variants (2.5%), copy-number variations (2.0%), frameshift insertions (1.5%), in-frame deletions and splice-site variants (1.0% each), and in-frame insertions (0.5%). Median VAF among sequence-level events with available data was 24.8% (IQR 15.1–36.1); VAF was interpreted descriptively because it is influenced by tumour cellularity, copy-number state, sample purity, and subclonality.

Sensitivity analyses are summarised in Supplementary Table S7.

**Figure 2:**
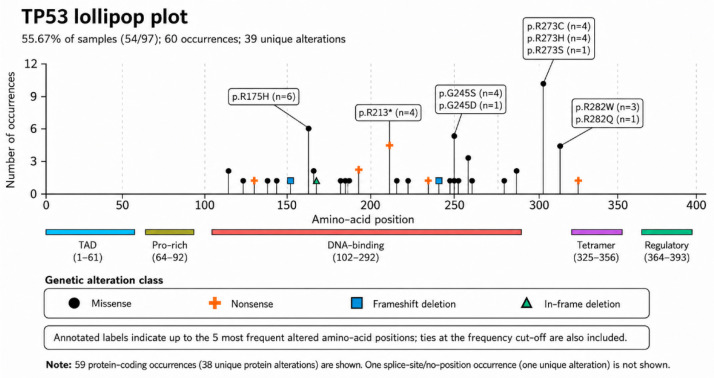
Lollipop plot of recurrent protein-level alterations in *TP53*. Protein-level alterations were distributed across the coding region, with recurrence at several canonical positions. An asterisk (*) denotes a nonsense variant introducing a premature stop codon at that position. One splice-site event without a protein coordinate is not represented. The plot is descriptive.

**Figure 3:**
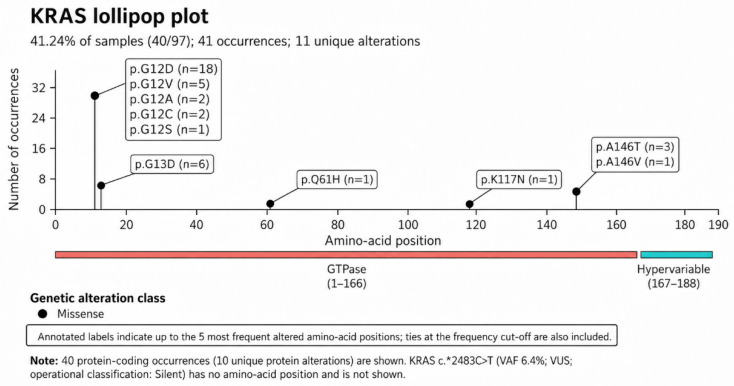
Lollipop plot of recurrent protein-level alterations in *KRAS*. Alterations were concentrated mainly at codons 12, 13, and 146, with p.G12D predominating.

**Figure 4:**
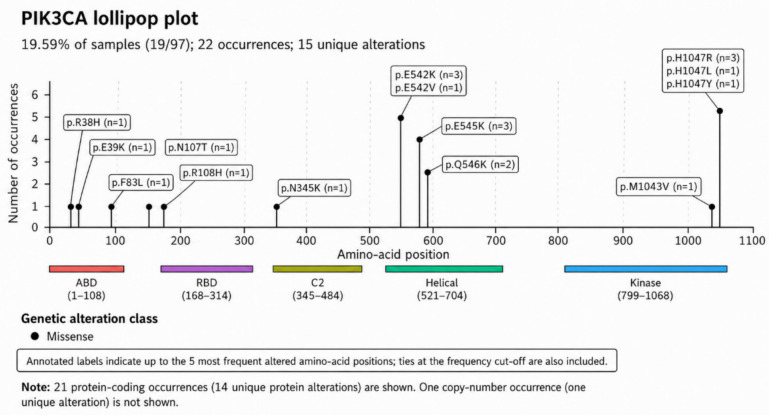
Lollipop plot of recurrent protein-level alterations in *PIK3CA*. Recurrent alterations involved the helical- and kinase-domain hotspot regions, including E542, E545, and H1047.

**Figure 5:**
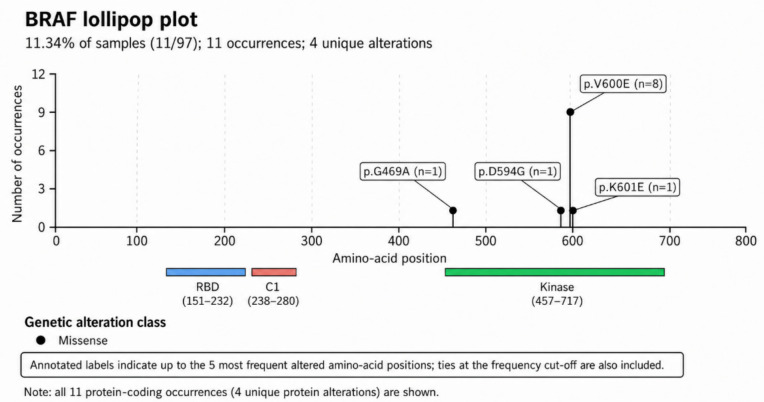
Lollipop plot of recurrent protein-level alterations in *BRAF*. Alterations were dominated by the canonical p.V600E hotspot.

Lollipop plots showed the expected contrast between hotspot-dominant oncogenes and the dispersed pattern of a tumour suppressor gene. *KRAS* alterations clustered mainly at codons 12, 13, and 146, with p.G12D predominating; *BRAF* was dominated by p.V600E; *TP53* alterations were distributed across the coding region; and *PIK3CA* showed recurrent E542, E545, and H1047 hotspots (Figure 2, Figure 3, Figure 4 and Figure 5). These plots are descriptive and include lollipop-representable reportable sequence-level events; 

### 2.7. Exploratory Molecular Associations to Predefined Clinical Presentation Features

The composite variable combining the predefined clinical phenotypes was not used as a molecular comparator because it encompasses biologically heterogeneous clinical scenarios. Molecular analyses were therefore organised according to five prespecified clinical features: CRC diagnosis at ≤50 years, significant polyposis, a malignant neoplasm preceding CRC, family history of CRC, and family history suggestive of CRC predisposition.

The three gene- and pathway-level comparisons with the lowest raw *p*-values for each phenotype are presented in Table 8 and Table 9 respectively. Analyses were restricted to pathogenic or likely pathogenic (P/LP) alterations, with gene-specific denominators reflecting valid assay coverage and pathway analyses based on the harmonised common-gene model.

**CRC diagnosis at ≤50 years.** Among the 31 patients diagnosed at ≤50 years, *NRAS* P/LP alterations were more frequent than among patients diagnosed after 50 years (3/31 versus 1/66; OR 6.96, 95% CI 0.69–69.89; raw *p* = 0.095). Conversely, *PIK3CA* and *KRAS* alterations were less frequent in the younger group. At pathway level, PI3K/AKT/mTOR involvement was observed in 4/31 versus 18/66 patients (OR 0.40, 95% CI 0.12–1.29; raw *p* = 0.115), followed by lower RAS/RAF/MAPK involvement and higher TP53/cell-cycle/checkpoint involvement.

**Significant polyposis.** Significant polyposis was present in four patients. The strongest gene-level contrasts involved *HRAS* (1/4 versus 0/93; OR 80.14, 95% CI 2.75–2339.46; raw *p* = 0.041), *BRAF* (2/4 versus 9/93; OR 9.33, 95% CI 1.17–74.49; raw *p* = 0.062), and *ERBB3* (1/4 versus 1/93; OR 30.67, 95% CI 1.53–616.52; raw *p* = 0.081). All four patients had RAS/RAF/MAPK pathway involvement, compared with 49/93 patients without polyposis. Receptor tyrosine kinase involvement was also more frequent, whereas PI3K/AKT/mTOR involvement was absent from the polyposis subgroup.

**Malignant neoplasm preceding CRC.** Seventeen patients had a malignant neoplasm preceding CRC. The leading pathway-level contrast was a lower frequency of PI3K/AKT/mTOR involvement (1/17 versus 21/80; OR 0.18, 95% CI 0.02–1.41; raw *p* = 0.108). Receptor tyrosine kinase involvement was more frequent, while RAS/RAF/MAPK involvement was similar between groups. At gene level, the highest-ranked comparisons involved *HRAS*, *PIK3CA*, and *CHEK2*, each based on a small number of alteration-positive patients.

**Family history of CRC.** After excluding patients with unknown family-history information, *BRAF* P/LP alterations were absent among patients with a family history of CRC and present in 9/47 patients with a documented negative history (0/17 versus 9/47; OR 0.12, 95% CI 0.01–2.10; raw *p* = 0.098). The other leading gene-level contrasts involved *CHEK2* and *ERBB2*. RAS/RAF/MAPK involvement was less frequent in the family-history-positive group (6/17 versus 25/47), while TP53/cell-cycle/checkpoint and PI3K/AKT/mTOR involvement showed smaller between-group differences.

**Family history suggestive of CRC predisposition.** *BRAF* P/LP alterations were likewise absent among patients with a family history suggestive of CRC predisposition and present in 9/49 patients with a documented negative history (0/15 versus 9/49; OR 0.14, 95% CI 0.01–2.51; raw *p* = 0.101). The next gene-level comparisons involved *CHEK2* and *ERBB2*. At pathway level, TP53/cell-cycle/checkpoint and RAS/RAF/MAPK involvement were less frequent in the predisposition-suggestive group, whereas G-protein/cAMP signalling was infrequent in both groups.

Sensitivity analyses in which patients with unknown family-history information were grouped with the negative category are presented in Supplementary Table S7.

Overall, the predefined clinical presentation features showed distinct gene- and pathway-level patterns, with the direction and magnitude of the observed associations varying across phenotypes. Following BH adjustment, these findings were classified as exploratory and are therefore reported as phenotype-specific molecular signals requiring independent validation.

### 2.8. Associations Between Predefined Clinical Presentation Features and Clinicopathological Characteristics

Clinicopathological comparisons were performed for the same five predefined clinical presentation features. The three variables with the lowest raw *p*-values for each feature are summarised in Table 10. Benjamini–Hochberg-adjusted values are provided to indicate the exploratory nature of these analyses.

**CRC diagnosis at ≤50 years.** Patients diagnosed at ≤50 years showed a different nodal-stage distribution from those diagnosed at an older age (raw *p* = 0.038), with relatively fewer N0 cases and a greater representation of N2 and NX categories. Differences were also observed in M- and T-stage distributions, although these were less pronounced (raw *p* = 0.059 and 0.110, respectively).

**Significant polyposis.** Significant polyposis was identified in four patients. Microsatellite instability was more frequent in this subgroup than in patients without significant polyposis (2/4 versus 16/90; OR 4.62, 95% CI 0.61–35.33; raw *p* = 0.164). All four patients were M0 at diagnosis, and three were classified as N0; however, comparisons of M- and N-stage distributions were based on very small numbers.

**Malignant neoplasm preceding CRC.** Patients with a malignant neoplasm preceding CRC had a nodal-stage distribution characterised by a higher proportion of N0 disease and fewer N1–N2 cases than patients without a previous malignancy (raw *p* = 0.080). Tumour budding was also less frequent in this group (4/13 versus 39/73; OR 0.39, 95% CI 0.11–1.37; raw *p* = 0.132). Sex distribution showed a smaller between-group difference.

**Family history of CRC.** After excluding patients with unknown family-history information, the clearest difference involved the pattern of perineural and vascular invasion (raw *p* = 0.009). Patients with a family history of CRC more frequently had no documented invasion and less frequently had combined perineural and vascular invasion. Tumour differentiation also differed between groups (raw *p* = 0.044), with a greater relative frequency of well-differentiated tumours in the family-history-positive group. Tumour budding was less frequent in these patients (4/15 versus 23/41; OR 0.28, 95% CI 0.08–1.04; raw *p* = 0.051).

**Family history suggestive of CRC predisposition.** A similar invasion pattern was observed in patients with a family history suggestive of CRC predisposition, including a higher frequency of absence of invasion and fewer cases with combined perineural and vascular invasion (raw *p* = 0.023). Tumour budding was less frequent in this group (3/13 versus 24/43; OR 0.24, 95% CI 0.06–0.99; raw *p* = 0.038). T-stage distribution also differed between groups (raw *p* = 0.048), with fewer T4 tumours among patients with a suggestive family history.

Overall, the predefined clinical features were accompanied by distinct clinicopathological patterns, particularly in nodal stage, invasion profile, tumour differentiation, and tumour budding, with the corresponding adjusted analyses (details on Supplementary Table S8) supporting their interpretation as exploratory findings.

### 2.9. Exploratory Principal Component Analysis and Multiple Correspondence Analysis

Exploratory principal component analysis (PCA) was performed using binary gene-level NGS variables to assess whether the overall molecular profile suggested discriminating separable patterns across clinical and pathological subgroups. Multiple correspondence analysis (MCA) was applied to the same matrix as a categorical sensitivity analysis.

The complete-case dimensional matrix included 97 patients and six shared genes meeting eligibility criteria: *NRAS*, *PIK3CA*, *BRAF*, *PTEN*, *TP53*, and *KRAS*.

**Figure 6:**
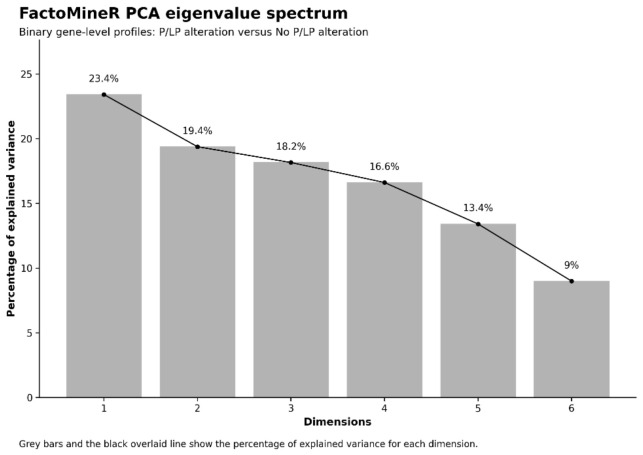
Explained variance of gene-level NGS principal components. Explained variance plot showing the proportion of total variance captured by each principal component in the gene-level NGS PCA. The moderate but still limited variance explained by the first two components indicates that the gene-level alteration matrix was sparse and multidimensional, and that two-dimensional PCA plots should be interpreted as exploratory visual summaries rather than evidence of discrete molecular clusters.

PCA showed that PC1 and PC2 explained 23.4% and 19.4% of the total variance, respectively (42.8% cumulatively), while the first five components accounted for 91.0% (Figure 6). MCA yielded the same uncorrected dimensional proportions. After applying the MCA-specific Benzécri correction, Dimensions 1–3 accounted for 82.6%, 13.4%, and 4.1% of the corrected inertia, respectively (Table 11; Supplementary Figure S1).

Patient-level PCA and MCA axes were perfectly correlated in absolute value (|r| = 1.000 for all matched axes), differing only by the expected constant scale factor and arbitrary sign reversals. Thus, MCA provided a categorical sensitivity representation of the same molecular geometry rather than a contradictory result. The direct correspondence between all six PCA and MCA axes is reported in Supplementary Table S9. The corresponding MCA representation of the same six-gene P/LP matrix is shown in Supplementary Figure S1.

**Figure 7:**
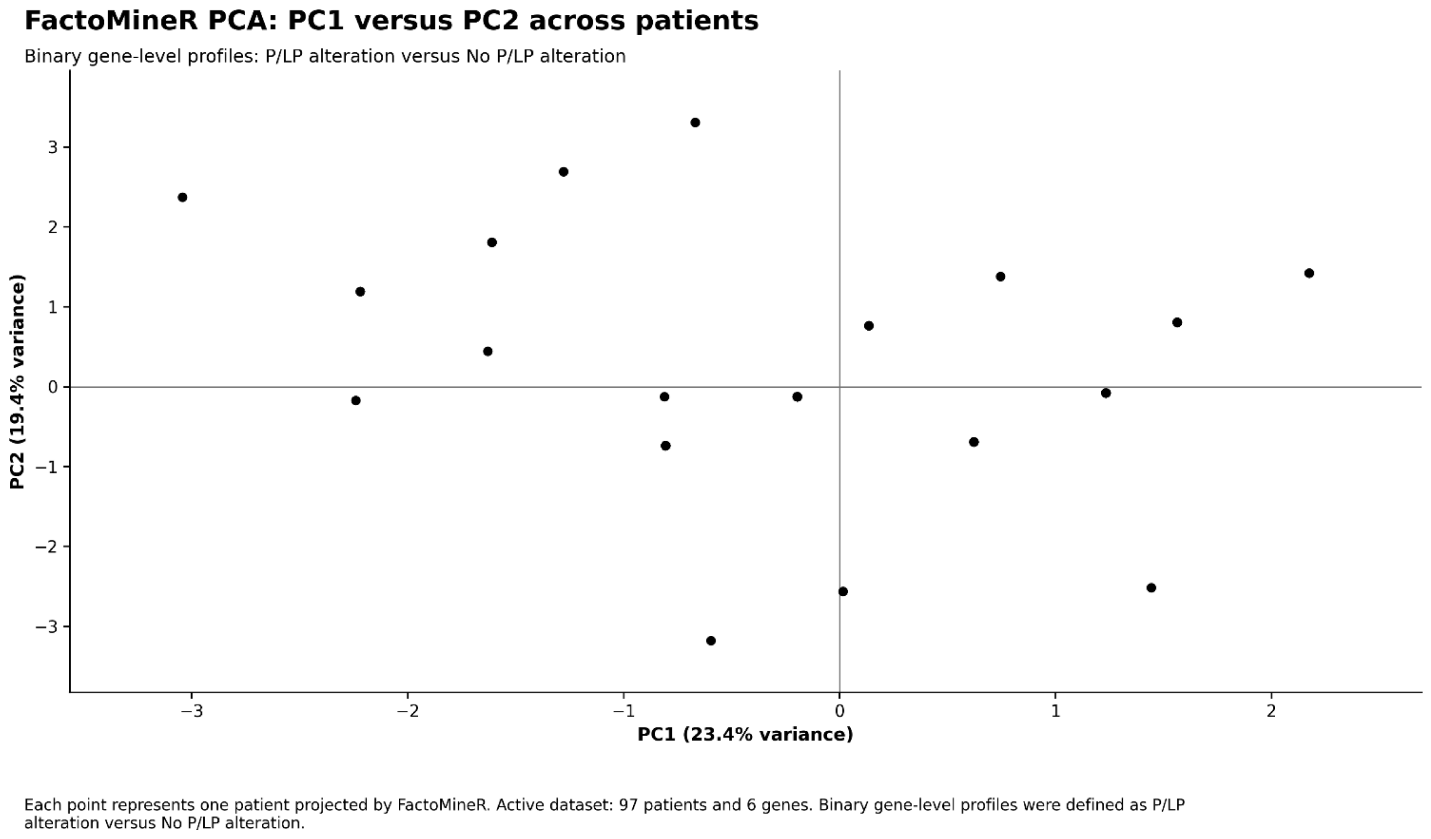
PCA of patient-level P/LP profiles. Proximity indicates similar molecular profiles; the overlapping central distribution and peripheral observations reflect a sparse, multidimensional structure rather than validated patient clusters.

**Figure 8:**
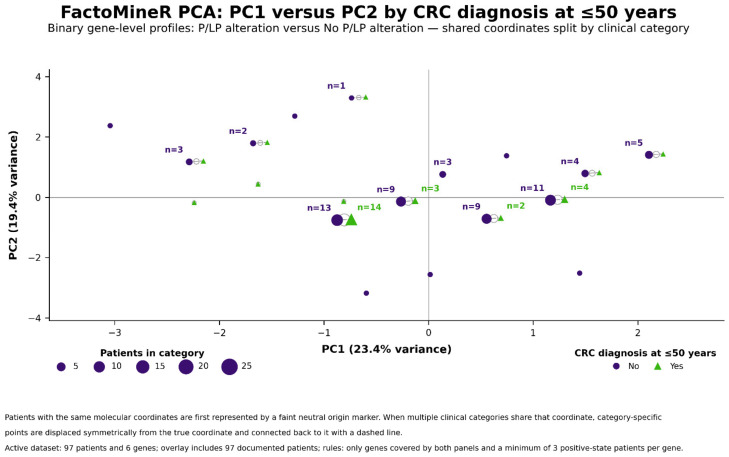
Overlap-aware FactoMineR PCA by early CRC diagnosis.

**Figure 9:**
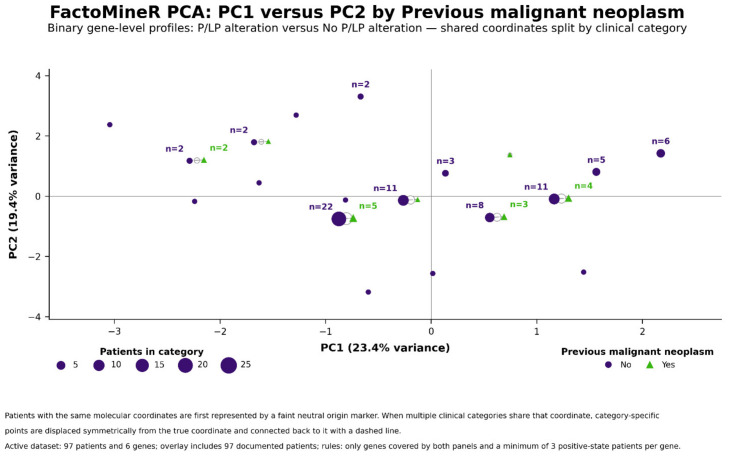
Overlap-aware FactoMineR PCA by previous malignant neoplasm.

**Figure 10:**
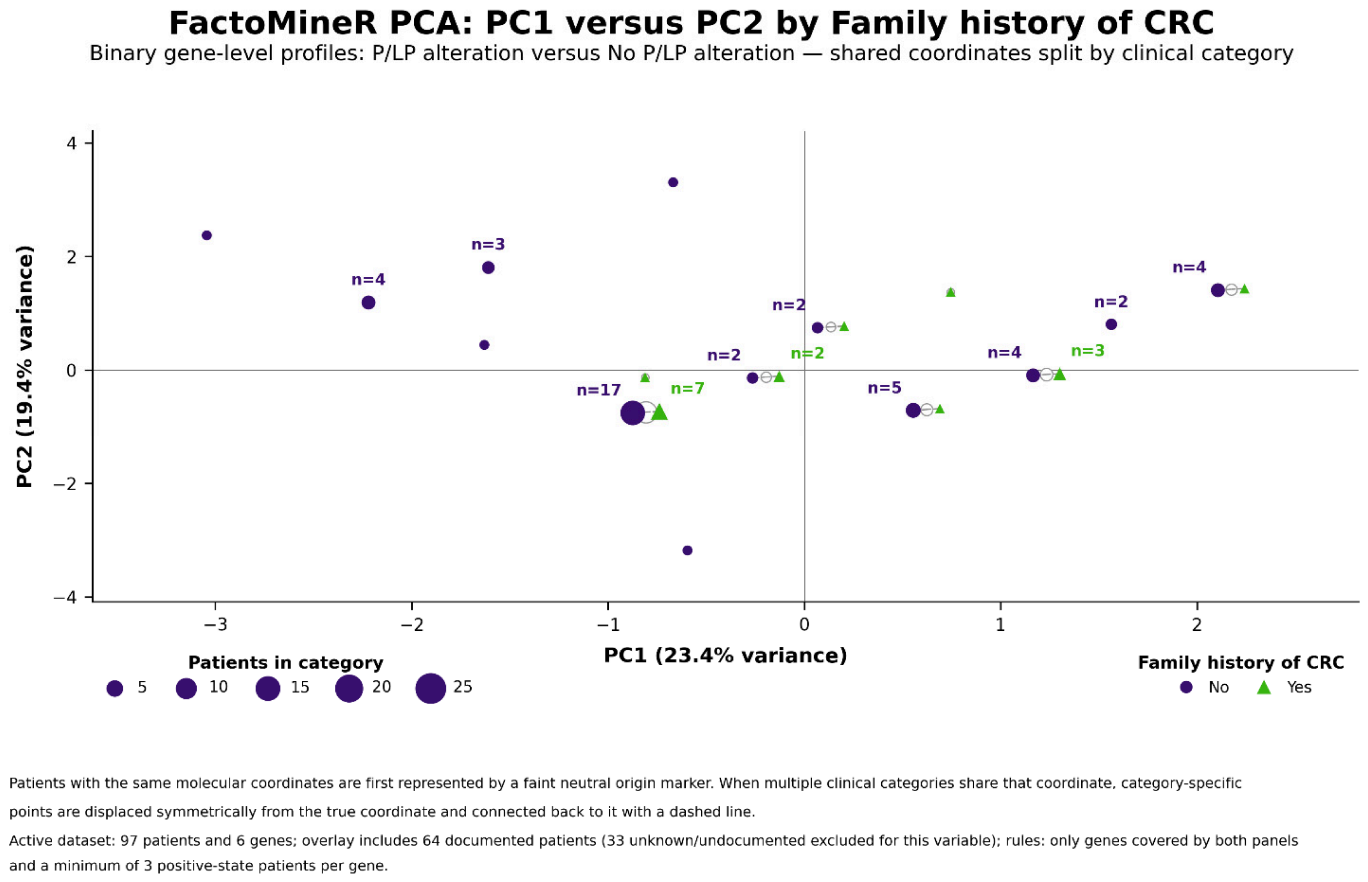
Overlap-aware FactoMineR PCA by family history of CRC.

**Figure 11:**
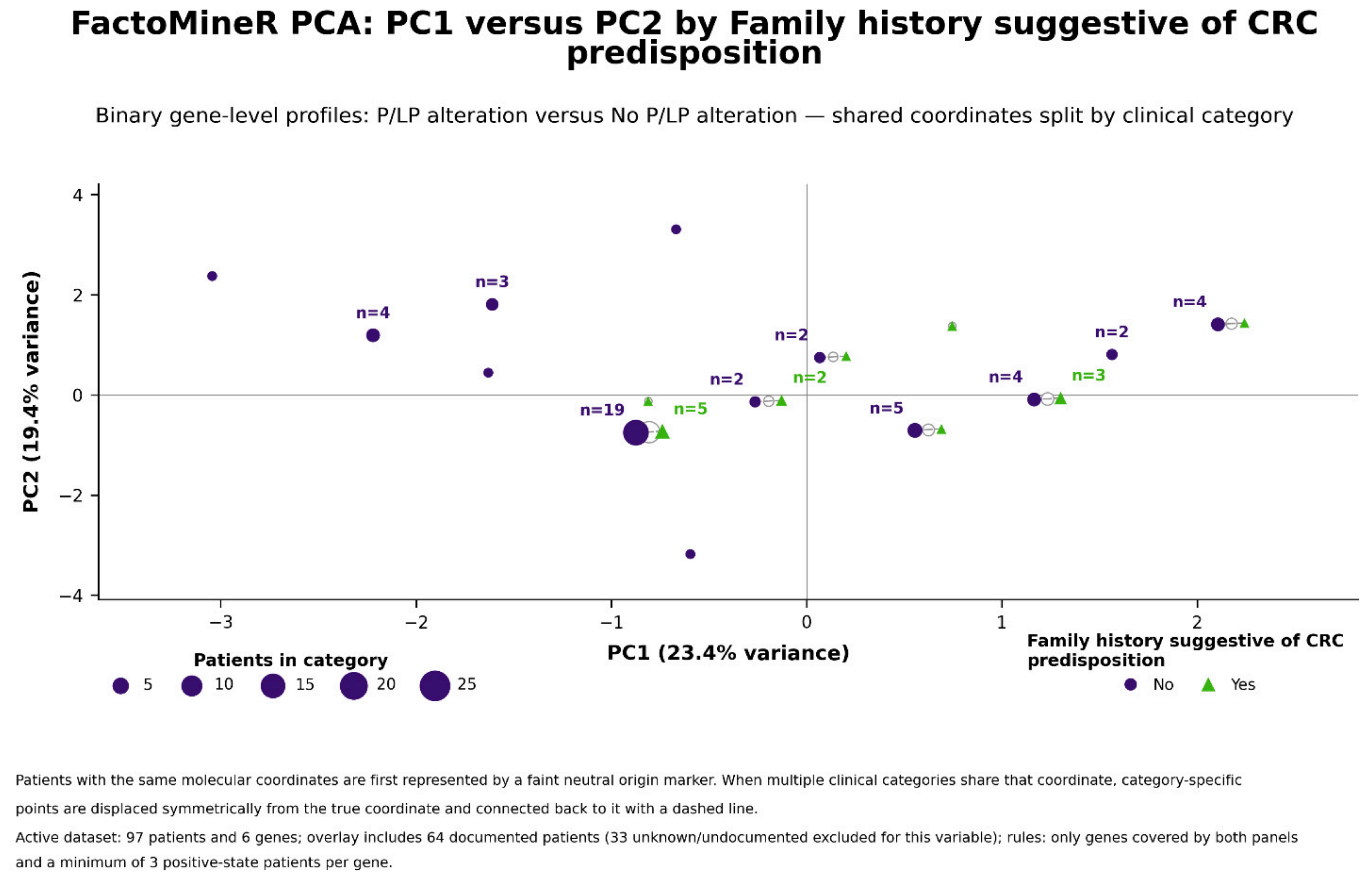
Overlap-aware FactoMineR PCA by family history suggestive of CRC predisposition.

No clear PCA-based separation was identified according to the formal predefined clinical features (Figure 7, Figure 8, Figure 9, Figure 10 and Figure 11).

Visual inspection of the PCA projection showed a sparse and multidimensional molecular structure. Most patients occupied an overlapping central region, whereas a smaller number of peripheral observations reflected less frequent combinations of P/LP alterations. No compact or clearly separated patient clusters were apparent. Instead, molecular variability was distributed across several principal components, with only partial and diffuse displacement of some clinical phenotype groups.

## 3. Discussion

This study characterised the molecular landscape detected by routine tumour-based targeted NGS and explored its relationship with five predefined clinical presentation features in patients with CRC. Reportable alterations were identified in 93.8% of patients, and 89.7% harboured at least one pathogenic or likely pathogenic alteration. The principal phenotype analyses identified selected gene-, pathway-, and clinicopathological patterns whose direction and magnitude varied across the clinical features examined. Benjamini–Hochberg adjustment classified these associations as exploratory rather than confirmatory; accordingly, they should be interpreted as focused hypotheses emerging from this cohort rather than disregarded because they did not meet an FDR-controlled threshold.

An additional strength of this study is the comprehensive molecular catalogue, including all pathogenic, likely pathogenic, VUS, silent, and copy-number variants, allowing complete transparency and facilitating future meta-analyses.

The overall molecular landscape was consistent with established CRC biology. *TP53*, *KRAS*, *PIK3CA*, and *BRAF* were the most frequently affected genes in the P/LP analysis, while TP53/cell-cycle regulation, RAS/RAF/MAPK signalling, and PI3K/AKT/mTOR signalling were the most frequently affected pathways. These findings are concordant with large molecular series of CRC and with the recognised contribution of cell-cycle disruption, MAPK activation, and PI3K pathway alterations to colorectal carcinogenesis [12,13,14,15,16,17,31,32,41]. Thus, although no group-specific signature was identified, the NGS dataset captured expected and interpretable CRC alterations.

The high proportion of patients with at least one reportable alteration supports the analytical yield of targeted NGS in this cohort. NGS can be more efficient than sequential single-gene testing when several biomarkers are relevant, because it assesses multiple genes and alteration classes from limited tumour material in a single workflow [26,28]. This is relevant in CRC, where *RAS*, *BRAF*, MSI/MMR status and selected rare targets may influence management in appropriate contexts [24,26,28]. Nevertheless, analytical yield should be distinguished from immediate therapeutic actionability, since the clinical relevance of an alteration depends on disease setting, alteration class, assay coverage, and available targeted strategies.

Among patients diagnosed with CRC at ≤50 years, *NRAS* P/LP alterations were more frequent, whereas *PIK3CA* and *KRAS* alterations and PI3K/AKT/mTOR pathway involvement were less frequent than in patients diagnosed at an older age. The younger group also showed a different nodal-stage distribution. These observations add to the heterogeneous literature on early-onset CRC, in which differences in sidedness, MSI/MMR status, precursor pathway, and selected driver alterations have varied between studies [8,9,10,11,42]. In a large comparison of early-onset and average-onset CRC, early-onset tumours were more commonly left-sided, but gene- and pathway-level differences were not retained after adjustment for tumour sidedness in microsatellite-stable disease [10]. Rather than indicating a uniform early-onset molecular subtype, the present findings describe selected molecular and clinicopathological directions that may help refine future analyses of this clinically diverse population.

Significant polyposis produced the most prominent gene-level molecular contrasts, although only four patients fulfilled this criterion. P/LP alterations in *HRAS*, *BRAF*, and *ERBB3* were more frequent in this subgroup, and all four patients had RAS/RAF/MAPK pathway involvement. The convergence of several alterations around related signalling mechanisms is noteworthy, despite the sparse counts and wide confidence intervals. Polyposis encompasses biologically different entities, including adenomatous and serrated polyposis, germline predisposition, mosaicism, low-penetrance susceptibility, and apparently sporadic polyposis [20,25,37,43]. Tumour-only hotspot panels cannot capture all of these mechanisms, but the observed pattern identifies a specific molecular hypothesis for evaluation in larger and more deeply characterised polyposis cohorts.

Patients with a malignant neoplasm preceding CRC showed a lower frequency of PI3K/AKT/mTOR pathway involvement. They also had a nodal-stage distribution with a greater representation of N0 disease and less frequent tumour budding. A previous malignancy is itself a heterogeneous clinical feature and may reflect age, surveillance intensity, shared environmental exposures, previous treatment, inherited susceptibility, or independent tumour occurrence. The molecular and pathological directions observed here should therefore be considered jointly, without assuming that they arise from a single underlying mechanism.

Family history of CRC was accompanied by selected molecular and pathological patterns. *BRAF* P/LP alterations were absent among patients with a documented positive family history, and RAS/RAF/MAPK pathway involvement was less frequent. At the pathological level, these patients showed a different distribution of perineural and vascular invasion, a higher relative frequency of well-differentiated tumours, and less frequent tumour budding. Family history is not equivalent to a defined hereditary syndrome and may result from high- or moderate-penetrance germline variants, polygenic susceptibility, shared exposures, or incomplete ascertainment [35,36]. The findings therefore describe a clinically defined familial phenotype rather than a genetically homogeneous subgroup.

Family history suggestive of CRC predisposition showed a partially similar pattern. *BRAF* alterations were absent in the positive group, while TP53/cell-cycle and RAS/RAF/MAPK pathway involvement were comparatively less frequent. This phenotype was also associated with differences in perineural and vascular invasion, tumour budding, and T-stage distribution. The recurrence of related pathological directions across the two family-history definitions provides internal coherence, while the differences between them also show that the precise operational definition of family history influences the resulting subgroup. Complete-case analyses excluding unknown records were therefore essential, although they reduced the available denominators.

Several factors may explain this result. First, the formal selected clinical features are clinically meaningful but biologically heterogeneous. Early diagnosis, family history, polyposis and previous malignancy may each reflect different combinations of inherited susceptibility, environmental exposures, tumour location, precursor pathway, surveillance intensity or stochastic tumour evolution. The same clinical phenotype label may therefore include patients with different underlying mechanisms. This is consistent with the concept that CRC comprises multiple clinicomolecular and precursor-lesion pathways rather than one homogeneous disease entity [8,16,17,21,29,40].

Second, tumour-only targeted NGS cannot capture all mechanisms underlying clinical presentations. The assays used here mainly evaluated reportable alterations within predefined genomic regions. They did not provide matched germline confirmation, genome-wide mutational signatures, methylation status, transcriptomic subtype, tumour microenvironment profiling or comprehensive structural variant assessment. This distinction is important because some of the best-established molecular classifications of CRC, including consensus molecular subtypes, rely heavily on transcriptomic, immune, stromal and epigenetic features rather than on hotspot mutation status alone [21]. Therefore, germline predisposition, polygenic risk, CIMP-related biology, serrated pathway epigenetics, immune contexture or non-panel alterations were likely not assessed or only indirectly represented.

Other key limitations include the retrospective single-centre design, modest cohort size, limited statistical power in rare-event subgroups, heterogeneous sequencing platforms, incomplete family-history documentation and absence of matched germline testing. Because NGS was requested according to routine clinical criteria rather than performed systematically in all CRC patients treated at the ULSSA, this cohort should be interpreted as a real-world NGS-profiled CRC cohort and not as an unselected CRC population. These limitations make false-negative findings possible and support an exploratory interpretation. A formal evaluation of specimen source as a determinant of the molecular findings was not undertaken given the small number of metastatic-biopsy samples (*n* = 2).

The PCA projections showed that the binary P/LP matrix had a sparse, overlapping, and multidimensional structure. The overlap-aware maps demonstrated that many patients shared a limited number of molecular coordinates, while peripheral observations represented less common combinations of alterations. No visually discrete phenotype-specific groups were apparent in the first two principal components. This should not be interpreted as evidence that the clinical phenotypes lack a biological basis; rather, it indicates that the six-gene binary matrix was not dominated by a single low-dimensional separation. MCA reproduced the same broad patient geometry when the data were treated categorically, supporting the stability of the visual configuration across analytical representations.

Clinical presentation features were accompanied by more apparent clinicopathological differences than by consistent targeted-NGS patterns, indicating that these features capture clinically relevant phenotypes that are only partially represented by the somatic alterations assessed in the present panels. In particular, CRC diagnosis at ≤50 years was associated with differences in stage distribution, whereas a malignant neoplasm preceding CRC and family-history-related variables were accompanied by selected pathological differences. Gene- and pathway-level analyses nevertheless identified phenotype-specific molecular directions, including RAS/RAF/MAPK-related signals in significant polyposis, lower PI3K/AKT/mTOR involvement in patients with a previous malignant neoplasm, and family-history-related differences involving *BRAF* and RAS/RAF/MAPK signalling. Together, these findings describe clinically meaningful but biologically heterogeneous phenotypes rather than clearly separated molecular subgroups defined by the targeted alterations assessed in this study.

Overall, targeted NGS provided high-yield molecular characterisation of CRC and identified focused phenotype-specific signals, although the molecular structure was not reducible to a single low-dimensional pattern in this cohort. The overlapping PCA distribution indicates that the predefined clinical presentations were not clearly separated by the six-gene binary P/LP matrix, but does not exclude molecular differences outside the genes, alteration classes, or biological layers assessed by the panels. The absence of statistically significant associations after correction for multiple testing should not be interpreted as evidence of absence of biological relationships. Rather, it reflects the exploratory nature of this single-centre cohort and the limited statistical power available for detecting modest genotype–phenotype associations.

That no association remained significant after multiple-testing correction is itself informative, reducing the likelihood of false-positive genotype–phenotype correlations frequently reported in small retrospective studies.

Our findings suggest a potential broader role for tumour molecular profiling as a complementary tool in CRC, beyond its established use in guiding targeted systemic therapies, pending validation of its direct clinical impact.

Future studies incorporating larger cohorts, systematic family-history assessment, paired germline testing, and broader molecular profiling strategies—including comprehensive genomic profiling, methylation analysis, transcriptomic classification, and circulating tumour DNA assessment—may validate the observed signals and support the development of integrated clinicopathological–molecular stratification models.

## 4. Materials and Methods

### 4.1. Study Design and Setting

This was a single-centre, retrospective observational study conducted at Centro Hospitalar Universitário de Santo António, Porto, Portugal. This study included patients with CRC who underwent molecular profiling using NGS as part of routine clinical care. No additional clinical assessments, imaging studies, laboratory tests, or biological sample collections were performed specifically for the purposes of this study.

Clinical, pathological and molecular data were retrieved retrospectively from institutional electronic medical records and molecular pathology reports. This study was designed to characterize tumour-based NGS profiles and identify patterns associated with predefined distinctive clinical presentation features in patients with CRC. Associations between molecular findings, clinicopathological variables and clinical outcomes were explored as secondary analyses.

Because NGS testing was performed according to routine clinical indications rather than systematically in all patients with CRC, the study population represents a real-world NGS-tested CRC cohort rather than an unselected institutional CRC population.

### 4.2. Study Population

This study comprised CRC patients followed at ULSSA. All patients had histologically confirmed CRC and had NGS testing performed on tumour tissue or metastatic biopsy material. Patients were included in this study irrespective of disease stage, provided that the NGS results and sufficient clinical information were available for analysis. The specific clinical indication for each NGS request could not be systematically abstracted from the institutional records, as this information was not consistently documented as a structured field in the molecular pathology reports.

**Figure 12:**
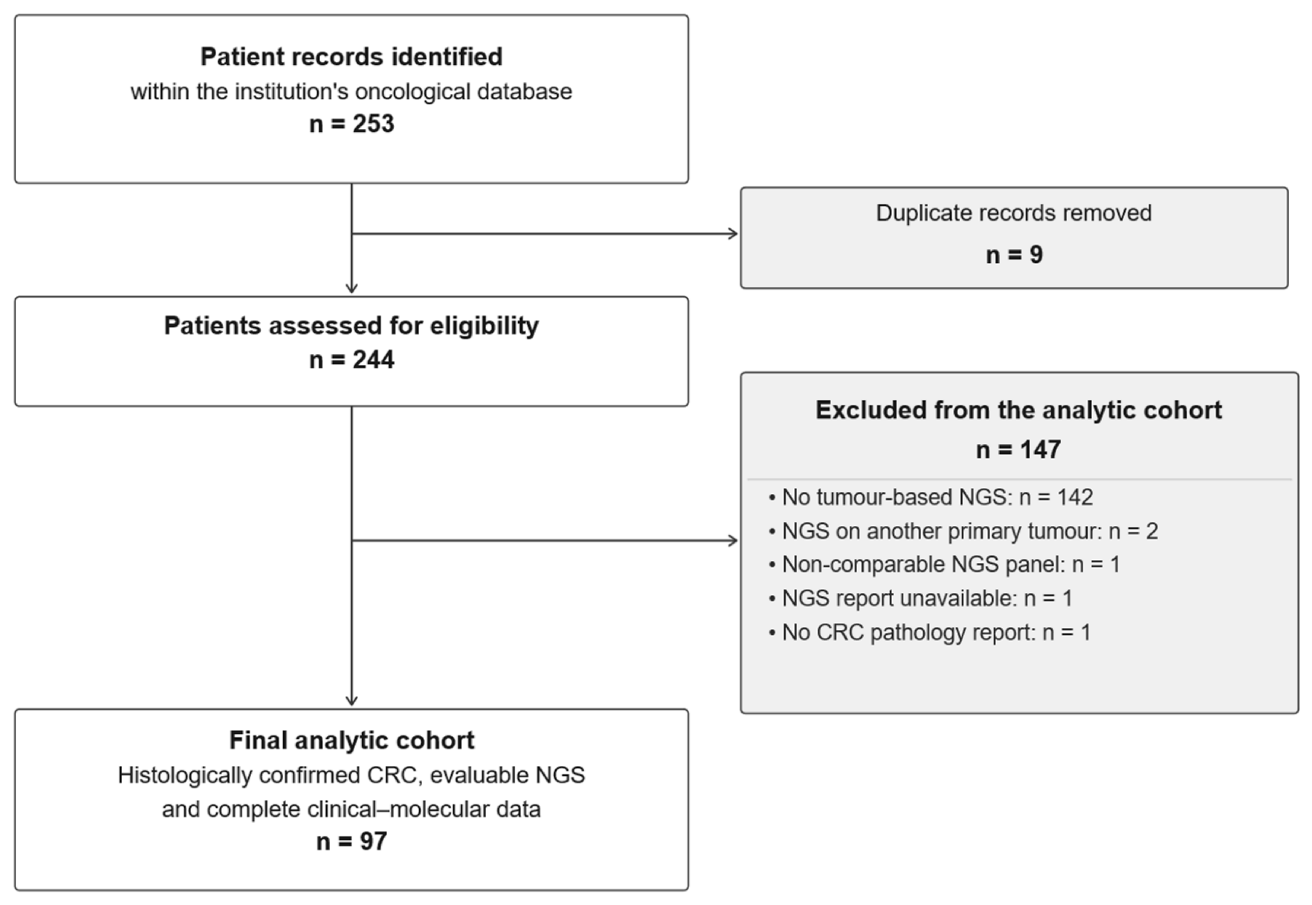
Flow diagram of patient identification, eligibility assessment, and inclusion in the final analytic cohort.

Patients with insufficient information for the CRC diagnosis or with inconclusive NGS data were excluded from the analytical cohort. Eligible patients were those who underwent NGS testing between January 2023 and the data cut-off of 31 May 2026, which corresponds to the database lock date for this analysis. Patient identification, eligibility assessment, and inclusion in the final analytic cohort are summarised in Figure 12.

### 4.3. Data Collection and Clinical Variables

Data were collected from electronic medical records, pathology reports, surgical records, oncology records, and molecular pathology reports. Extracted variables included demographic data and age at diagnosis. Specifically for CRC: tumour site and anatomical location, histological type, tumour differentiation, vascular and/or perineural invasion, tumour budding, microsatellite instability (MSI) or mismatch repair (MMR) status when available, TNM stage (according to the AJCC 8th edition), treatment, recurrence, and clinical status at last follow-up.

TNM stage was recorded at diagnosis, metastatic-site variables were analysed cumulatively, including metastases present at diagnosis and those documented later during follow-up. Metastatic-site categories were not mutually exclusive.

For colorectal polyps, the variables collected included presence and number of polyps, histological type and highest grade of dysplasia when available. Colorectal polyps, including those with high-grade dysplasia or adenocarcinoma when documented in clinical records, were not analysed as independent index lesions or separate molecular entities. Polyp data were used only to define polyp burden and significant polyposis.

Family-history variables were extracted as documented in the clinical record and included family history of cancer, family history of CRC, cancer in first-degree relatives and family-history patterns suggestive of CRC predisposition. Unknown or undocumented family-history information was retained as unknown and was not assumed to be negative.

### 4.4. NGS and Molecular Data Handling

NGS was performed as part of routine clinical care on tumour tissue or metastatic biopsy material. Molecular data were retrieved from the original clinical molecular pathology reports. No sequencing was performed specifically for this study.

For the NGS studies, two methodologies were employed: either amplicon-based sequencing using the Genexus platform (ThermoFisher Scientific, Waltham, MA, USA) and employing the Oncomine Precision Assay (OPA) ≈50-gene panel or hybrid-capture-based sequencing using the MiSeq platform (Illumina, San Diego, CA, USA) and a custom-made ≈40-gene panel (Qiagen, Hilden, Germany); please see Supplementary Table S10. All DNA-based NGS testing involved gene hotspots. The NGS panels used did not provide whole-gene coverage of the genes studied. Gene expression was not quantified, and no differential gene-expression analysis was performed. Although two sequencing platforms were used during the study period, all reported variants underwent identical clinical interpretation according to institutional molecular pathology procedures.

NGS was performed on FFPE specimens obtained at diagnosis or surgical resection, comprising surgical resection specimens (82/97), pre-operative biopsies (13/97), and metastatic lesion biopsies (2/97); full specimen-level characteristics are detailed in Supplementary Table S11.

A reportable molecular alteration was defined as any gene-level alteration recorded in the original clinical molecular pathology report and abstracted into the study database. All reportable alteration events were used for descriptive landscape analyses. For principal gene-, pathway-, PCA-, and MCA-based inference, sequence-level alterations classified as pathogenic or likely pathogenic (P/LP) were retained. VUS non-silent sequence-level events, silent variants, and copy-number variations were tabulated separately and were not included in the principal P/LP comparisons. 

MSI status assessed by PCR and/or MMR protein expression assessed by immunohistochemistry were abstracted from pathology reports when available and were not treated as NGS-derived variables.

For analytical purposes, alterations were categorized by gene, alteration type when available, and affected molecular pathway. Molecular pathways were defined *a priori* according to known functional relationships and are listed in Table 12.

Pathway categories were investigator-defined and were not mutually exclusive. For descriptive pathway-level summaries, a pathway was considered affected when at least one P/LP alteration was detected in a covered gene assigned to that category. Genes not covered by the assay used were treated as untested. Pooled inferential pathway comparisons were restricted to P/LP alterations in genes shared by both platforms. These categories reflect the analysed gene groups and should not be interpreted as comprehensive pathway activation status.

Because testing was performed on tumour material or metastatic biopsy material without matched germline analysis, all detected alterations were interpreted as somatic findings. Germline origin could neither be established nor excluded. The absence of a reported alteration was interpreted as absence of a reportable alteration within the genomic regions covered by the assay used, and not as absence of any genomic alteration in that gene or tumour.

### 4.5. Definition of Groups with Distinctive Clinical Presentation Features

Groups of distinctive clinical presentation features were defined before molecular association analyses and independently of NGS results, MSI/MMR status, or any other molecular findings. This was done to avoid circularity between clinical subgroup definition and molecular outcomes.

The definition criteria are documented in Table 13.

The clinical presentation features were not mutually exclusive, and individual patients could fulfil criteria for more than one subgroup.

Unknown or undocumented information was not considered positive unless the clinical record explicitly documented that feature. For family-history variables, primary analyses excluded unknowns. Sensitivity analyses grouped unknowns with the negative category.

For descriptive purposes, patients presenting at least one predefined feature were classified as having a distinctive clinical presentation profile. However, primary exploratory analyses focused on individual clinical features rather than the composite variable due to biological heterogeneity.

### 4.6. Statistical Analysis

Continuous variables were summarised as mean ± standard deviation, and categorical variables as absolute and relative frequencies. Denominators reflected available valid data. Associations between prespecified clinical features and categorical outcomes were assessed using Pearson chi-square tests or Fisher exact tests, as appropriate. Odds ratios with 95% confidence intervals were calculated for 2 × 2 comparisons when estimable. Multicategorical outcomes were assessed using global Pearson 2 × K tests.

This study was exploratory. Raw *p*-values, effect estimates, direction, biological plausibility, and consistency across related analyses were retained for hypothesis generation. BH adjustment was applied within six prespecified analytical families: three families for the five principal phenotypes (P/LP genes, harmonised P/LP pathways, and biological clinicopathological variables) and the corresponding three families for family-history sensitivity analyses. Adjusted *p* < 0.05 defined confirmatory FDR-controlled evidence. Results with raw *p* < 0.05 but adjusted *p* ≥ 0.05 were reported as nominal exploratory signals and were not interpreted as biologically null solely because they did not meet the confirmatory threshold.

PCA was performed on a standardised binary P/LP common-gene matrix. Genes were retained when shared by both platforms, completely observed, with at least 3 distinct alterations, producing a 97-patient × 6-gene matrix. MCA was applied to the same matrix as a categorical sensitivity analysis.

Analyses were performed using Microsoft Excel for Microsoft 365, IBM SPSS Statistics version 31.0.2.0, and R version 4.5.3. Oncoplots and lollipop plots were generated using maftools (2.26.1) [44]. PCA and MCA were implemented using standard R and FactoMineR-compatible workflows (version 2.16).

### 4.7. Ethical Considerations

This study was approved by the institutional bodies of ULSSA and ICBAS, including the Ethics Committee, under approval/reference number 2025.269 (227-CAC-227-CE), approval date on 31 March 2026. Data collection was retrospective and based on clinical and molecular data obtained during routine care. No additional consultations, diagnostic procedures, imaging studies, laboratory tests, or biological sample collections were performed for this study. Data were recorded in anonymised form, and confidentiality of participants’ identity and clinical information was ensured.

## 5. Conclusions

In this real-world cohort of patients with CRC undergoing routine tumour-based NGS, most cases harboured at least one reportable molecular alteration. The molecular landscape was dominated by expected CRC genes and pathways, particularly *TP53*, *KRAS*, *PIK3CA*, *BRAF*, TP53/cell-cycle regulation, and *RAS*/*RAF*/*MAPK* signalling, supporting the biological plausibility and descriptive molecular value of targeted sequencing in routine clinical practice.

Although selected associations between molecular alterations and predefined clinical presentation features were observed, these findings were generally infrequent, driven by low-frequency events and not consistently reproducible across analyses. No robust molecular signature was identified for early age at diagnosis, significant polyposis, personal history of another malignant neoplasm, or family-history-related clinical features. Likewise, exploratory PCA analyses did not support the existence of discrete molecular clusters corresponding to these clinical presentations.

These findings support the use of targeted NGS as a complementary molecular characterisation tool, while highlighting the need for broader integrated clinicopathological-molecular models to explain heterogeneous clinical presentations in CRC.

## Figures and Tables

**Table 1:** General characteristics of the total cohort.

Variable	Total Cohort, *n* = 97
Total patients with NGS performed	97
Follow-up time, months, mean ± SD	17.36 ± 13.6
**Sex**	
Female	45 (46.4%)
Male	52 (53.6%)
**Clinical status at last follow-up**	
Alive without disease	48 (49.5%)
Alive with disease	39 (40.2%)
Disease-related death	10 (10.3%)
**Primary tumour**	
Colon	73 (75.3%)
Rectum	24 (24.7%)
**Polyp burden**	
No polyps documented	37 (38.1%)
<10 polyps	34 (35.1%)
≥10 and <20 polyps	2 (2.1%)
≥20 and <100 polyps	2 (2.1%)
No colonoscopy	22 (22.7%)

Values are presented as *n* (%) unless otherwise specified. Percentages were calculated using the total NGS cohort. Follow-up is shown as mean ± standard deviation. NGS, next-generation sequencing; SD, standard deviation.

**Table 2:** Clinicopathological characteristics of patients with CRC.

(**A**). **Tumour characteristics and staging**					
Age at diagnosis, years, mean ± SD	63.2 ± 13.1	**Perineural/vascular invasion**	*n* = 87	**T stage**	***n* = 96**
		No invasion	30 (34.5%)	T0	1 (1.0%)
		Perineural invasion only	10 (11.5%)	T1	10 (10.4%)
		Vascular invasion only	20 (23.0%)	T2	8 (8.3%)
		Both perineural and vascular invasion	27 (31.0%)	T3	53 (55.2%)
**Primary tumour site**	***n* = 97**	Missing	10	T4	21 (21.9%)
Colon	73 (75.3%)	**Tumour budding**	***n* = 86**	TX	3 (3.1%)
Rectum	24 (24.7%)	Present	43 (50.0%)	Missing	1
**Histological type**	***n* = 97**	Absent	43 (50.0%)	**N stage**	***n* = 96**
Adenocarcinoma	90 (92.8%)	Missing	11	N0	41 (42.7%)
Squamous cell carcinoma	5 (5.2%)	**Tumour differentiation**	***n* = 82**	N1	28 (29.2%)
Other	2 (2.1%)	Well differentiated	16 (19.5%)	N2	19 (19.8%)
**Tumour location**	***n* = 97**	Moderately differentiated	60 (73.2%)	N+	2 (2.1%)
Right colon	34 (35.1%)	Poorly differentiated	4 (4.9%)	NX	6 (6.2%)
Transverse colon	10 (10.3%)	Undifferentiated	2 (2.4%)	Missing	1
Left colon	16 (16.5%)	Missing	15	**M stage**	***n* = 96**
Sigmoid colon	26 (26.8%)	**Microsatellite instability**	***n* = 94**	M0	74 (77.1%)
Rectosigmoid junction	3 (3.1%)	MSI detected	18 (19.1%)	M1	20 (20.8%)
Rectum	19 (19.6%)	No MSI detected	76 (80.9%)	MX	2 (2.1%)
		Missing	3	Missing	1
(**B**). **Metastatic sites, later metastasis/recurrence and treatment**					
**Metastatic sites**	***n* = 96**	**Treatment**	***n*/*N* (%)**		
No metastasis recorded	60 (62.5%)	Surgery performed	89/96 (92.7%)		
Liver	22 (22.9%)	No surgery	7/96 (7.3%)		
Lung	14 (14.6%)	Palliative CTx	21/97 (21.6%)		
Peritoneum	10 (10.4%)	Palliative RT	4/97 (4.1%)		
CNS	0 (0.0%)	Adjuvant CTx	36/97 (37.1%)		
Other	9 (9.4%)	Neoadjuvant CTx	8/97 (8.2%)		
Missing	1	Neoadjuvant RT	7/97 (7.2%)		
**Subsequent metastasis/recurrence**	***n* = 95**	**New malignant neoplasm**	***n* = 97**		
No	74 (77.9%)	No	89 (91.8%)		
Subsequent metastasis	19 (20.0%)	Yes	8 (8.2%)		
Recurrence	3 (3.2%)				
Missing	2				

Values are presented as *n* (%) unless otherwise specified. Percentages use the valid denominators shown in the variable label when applicable. T, N, and M stage refer to staging at diagnosis. Metastatic-site variables were recorded cumulatively during the disease course, including metastases present at diagnosis and those documented during follow-up; metastatic sites and treatment categories were not mutually exclusive. Subsequent metastasis/recurrence was not mutually exclusive. CNS, central nervous system; MSI, microsatellite instability; SD, standard deviation; CTx, chemotherapy; RT, radiotherapy.

**Table 3:** Family history data in the total NGS cohort.

Variable	No	Yes	Unknown
Any family history of cancer	20 (20.6%)	43 (44.3%)	34 (35.1%)
Family history of CRC	47 (48.5%)	17 (17.5%)	33 (34.0%)
Cancer in a first-degree relative	31 (32.0%)	29 (29.9%)	37 (38.1%)
Family history suggestive of hereditary CRC	49 (50.5%)	15 (15.5%)	33 (34.0%)

**Table 4:** Distribution of predefined clinical presentation features.

Variable	Total Cohort, *n* = 97
Usual presentation	46 (47.4%)
At least one selected clinical feature	51 (52.6%)
**Selected clinical feature criteria**	
CRC diagnosed at ≤50 years †	31 (32.0%)
Significant polyposis, ≥10 polyps	4 (4.1%)
Previous malignant neoplasm	17 (17.5%)
Family history of CRC †	17 (17.5%)
Family history suggestive of CRC predisposition †	15 (15.5%)

Values are presented as *n* (%). The selected clinical feature criteria were not mutually exclusive. CRC, colorectal cancer. † Variable used for inferential analysis excluded patients with unknown family members.

**Table 5:** Global NGS findings.

**A. Assay and analytical yield**	***n* = 97**
**NGS method performed**	
Oncomine	87 (89.7%)
MiSeq	10 (10.3%)
**Any reportable NGS alteration detected**	
Yes	91 (93.8%)
No reportable alteration detected	6 (6.2%)
**Any P/LP alteration**	
Yes	87 (89.7%)
No P/LP alteration detected	10 (10.3%)
**B. Number of genes with P/LP alterations**	
0	10 (10.3%)
1	43 (44.3%)
2	29 (29.9%)
3	13 (13.4%)
4	1 (1.0%)
5	1 (1.0%)
**C. Number of affected molecular pathways per patient (P/LP)**	
0	10 (10.3%)
1	44 (45.4%)
2	31 (32.0%)
3	10 (10.3%)
4	1 (1.0%)
5	1 (1.0%)

Values are presented as *n* (%). “No reportable alteration detected” refers to absence of reportable alterations in the available NGS data. NGS, next-generation sequencing; P/LP, pathogenic or likely pathogenic.

**Table 6:** Descriptive gene-level NGS findings for genes with at least three unique reportable alterations.

Gene	Total Genetic Alterations, n	Unique Genetic Alterations, n	Patients with ≥1 P/LP Alteration, *n*/*N* Tested (%)	VUS Alteration Events, n	CNV Events, n	Silent Alteration Events, n
*TP53*	60	39	54/97 (55.7%)	0	0	0
*KRAS*	41	11	39/97 (40.2%)	0	0	1
*PIK3CA*	22	15	18/97 (18.6%)	1	1	0
*BRAF*	11	4	11/97 (11.3%)	0	0	0
*ERBB4*	7	3	0/97 (0.0%)	7	0	0
*ERBB2*	6	6	2/97 (2.1%)	3	0	1
*PTEN*	5	5	3/97 (3.1%)	0	1	0
*NRAS*	4	4	4/97 (4.1%)	0	0	0
*ALK*	3	3	0/97 (0.0%)	2	0	1
*FBXW7*	3	3	2/10 (20.0%)	1	0	0

P/LP, pathogenic or likely pathogenic; VUS, variant of uncertain significance; CNV, copy-number variation. Total genetic alterations correspond to all reportable alteration events, irrespective of classification. Unique genetic alterations correspond to distinct alteration identities after recurrent observations in different patients were collapsed. The P/LP column is patient-based and uses the number of patients with valid gene coverage as the denominator.

**Table 7:** Descriptive pathway-level NGS findings based on P/LP alterations across all genes covered by either platform.

Molecular Pathway	Patients with ≥1 P/LP Alteration, *n*/*N* (%)
TP53/cell-cycle regulation	56/97 (57.7%)
RAS/RAF/MAPK signalling	53/97 (54.6%)
PI3K/AKT/mTOR signalling	22/97 (22.7%)
Receptor tyrosine kinase signalling	5/97 (5.2%)
G-protein signalling	3/97 (3.1%)
DNA damage repair/polymerase proofreading	2/97 (2.1%)
Nuclear receptor signalling	1/97 (1.0%)
Other non-canonical/exploratory alterations	1/97 (1.0%)
WNT/β-catenin signalling	1/97 (1.0%)
TGF-β/SMAD signalling	1/10 (10.0%)
Hedgehog signalling	0/87 (0.0%)

A pathway was considered affected when a patient harboured at least one P/LP alteration in any constituent gene covered by the corresponding NGS platform. Patients were counted once within each pathway, and denominators were adjusted according to gene coverage. Untested genes were not considered unaltered.

**Table 8:** Top three individual gene-level P/LP comparisons for each prespecified clinical feature.

Clinical Feature	Gene	Comparison	OR (95% CI)	Raw *p*	BH-Adjusted *p*
CRC diagnosis at ≤50 years	*NRAS*	3/31 vs. 1/66	6.96 (0.69–69.89)	0.095	1.000
	*PIK3CA*	3/31 vs. 15/66	0.36 (0.10–1.37)	0.123	1.000
	*KRAS*	9/31 vs. 30/66	0.49 (0.20–1.23)	0.124	1.000
Significant polyposis	*HRAS*	1/4 vs. 0/93	80.14 (2.75–2339.46)	0.041	1.000
	*BRAF*	2/4 vs. 9/93	9.33 (1.17–74.49)	0.062	1.000
	*ERBB3*	1/4 vs. 1/93	30.67 (1.53–616.52)	0.081	1.000
Malignant neoplasm preceding CRC	*HRAS*	1/17 vs. 0/80	14.64 (0.57–375.29)	0.175	1.000
	*PIK3CA*	1/17 vs. 17/80	0.23 (0.03–1.87)	0.183	1.000
	*CHEK2*	1/17 vs. 0/70	12.82 (0.50–329.02)	0.195	1.000
Family history of CRC †	*BRAF*	0/17 vs. 9/47	0.12 (0.01–2.10)	0.098	1.000
	*CHEK2*	1/14 vs. 0/41	9.22 (0.35–240.01)	0.255	1.000
	*ERBB2*	1/17 vs. 0/47	8.64 (0.34–222.58)	0.266	1.000
Family history suggestive of CRC predisposition †	*BRAF*	0/15 vs. 9/49	0.14 (0.01–2.51)	0.101	1.000
	*CHEK2*	1/12 vs. 0/43	11.35 (0.43–297.38)	0.218	1.000
	*ERBB2*	1/15 vs. 0/49	10.24 (0.40–265.12)	0.234	1.000

P/LP, pathogenic or likely pathogenic; OR, odds ratio; CI, confidence interval; BH, Benjamini–Hochberg. The three gene-level comparisons with the lowest raw *p*-values are shown for each clinical feature. Comparisons indicate patients with versus without the feature. Gene-specific denominators reflect valid assay coverage. † Patients with unknown information were excluded from the analysis. BH-adjusted *p*-values indicate the exploratory nature of the findings.

**Table 9:** Top three individual pathway comparisons for each prespecified clinical feature.

Clinical Feature	Pathway	Comparison	OR (95% CI)	Raw *p*	BH-Adjusted *p*
CRC diagnosis at ≤50 years	PI3K/AKT/mTOR	4/31 vs. 18/66	0.40 (0.12–1.29)	0.115	1.000
	RAS/RAF/MAPK	14/31 vs. 39/66	0.57 (0.24–1.35)	0.199	1.000
	TP53/cell-cycle/checkpoint	20/31 vs. 34/66	1.71 (0.71–4.13)	0.229	1.000
Significant polyposis	RAS/RAF/MAPK	4/4 vs. 49/93	8.09 (0.42–154.54)	0.124	1.000
	Receptor tyrosine kinase signalling	1/4 vs. 4/93	7.42 (0.62–88.12)	0.194	1.000
	PI3K/AKT/mTOR	0/4 vs. 22/93	0.35 (0.02–6.81)	0.571	1.000
Malignant neoplasm preceding CRC	PI3K/AKT/mTOR	1/17 vs. 21/80	0.18 (0.02–1.41)	0.108	1.000
	Receptor tyrosine kinase signalling	2/17 vs. 3/80	3.42 (0.53–22.27)	0.210	1.000
	RAS/RAF/MAPK	10/17 vs. 43/80	1.23 (0.43–3.55)	0.703	1.000
Family history of CRC †	RAS/RAF/MAPK	6/17 vs. 25/47	0.48 (0.15–1.51)	0.206	1.000
	TP53/cell-cycle/checkpoint	10/17 vs. 31/47	0.74 (0.24–2.30)	0.599	1.000
	PI3K/AKT/mTOR	3/17 vs. 11/47	0.70 (0.17–2.90)	0.742	1.000
Family history suggestive of CRC predisposition †	TP53/cell-cycle/checkpoint	8/15 vs. 33/49	0.55 (0.17–1.80)	0.322	1.000
	RAS/RAF/MAPK	6/15 vs. 25/49	0.64 (0.20–2.07)	0.455	1.000
	G-protein/cAMP signalling	0/15 vs. 1/49	1.04 (0.04–26.94)	1.000	1.000

P/LP, pathogenic or likely pathogenic; OR, odds ratio; CI, confidence interval; BH, Benjamini–Hochberg. The three pathway-level comparisons with the lowest raw *p*-values are shown for each clinical feature. Pathway involvement was defined by at least one P/LP alteration in the harmonised common-gene model. † Patients with unknown information were excluded from the analysis. BH-adjusted *p*-values indicate the exploratory nature of the findings.

**Table 10:** Top three clinicopathological comparisons for each prespecified clinical feature.

Clinical Feature	Variable	Comparison	OR (95% CI)	Raw *p*	BH-Adjusted *p*
CRC diagnosis at ≤50 years	N stage at diagnosis	≤50 years: N0 = 10, N1 = 7, N2 = 8, N+ = 1, NX = 5; >50 years: N0 = 31, N1 = 21, N2 = 11, N+ = 1, NX = 1	Not applicable	0.038	0.328
	M stage at diagnosis	≤50 years: M0 = 25, M1 = 4, MX = 2; >50 years: M0 = 49, M1 = 16, MX = 0	Not applicable	0.059	0.333
	T stage at diagnosis	≤50 years: T0 = 0, T1 = 2, T2 = 1, T3 = 18, T4 = 7, TX = 3; >50 years: T0 = 1, T1 = 8, T2 = 7, T3 = 35, T4 = 14, TX = 0	Not applicable	0.110	0.451
Significant polyposis	Microsatellite instability	MSI detected: 2/4 vs. 16/90	4.62 (0.61–35.33)	0.164	0.528
	M stage at diagnosis	Polyposis: M0 = 4, M1 = 0, MX = 0; no polyposis: M0 = 70, M1 = 20, MX = 2	Not applicable	0.538	0.933
	N stage at diagnosis	Polyposis: N0 = 3, N1 = 0, N2 = 1, N+ = 0, NX = 0; no polyposis: N0 = 38, N1 = 28, N2 = 18, N+ = 2, NX = 6	Not applicable	0.619	0.933
Malignant neoplasm preceding CRC	N stage at diagnosis	Previous malignancy: N0 = 11, N1 = 3, N2 = 1, N+ = 1, NX = 0; no previous malignancy: N0 = 30, N1 = 25, N2 = 18, N + = 1, NX = 6	Not applicable	0.080	0.359
	Tumour budding	Present: 4/13 vs. 39/73	0.39 (0.11–1.37)	0.132	0.496
	Sex	Male: 11/17 vs. 41/80	1.74 (0.59–5.17)	0.312	0.845
Family history of CRC †	Perineural/vascular invasion	Family history: no invasion = 9, perineural only = 0, vascular only = 4, both = 2; negative history: no invasion = 8, perineural only = 8, vascular only = 8, both = 17	Not applicable	0.009	0.328
	Tumour differentiation	Family history: well = 5, moderate = 7, poor = 2, undifferentiated = 0; negative history: well = 4, moderate = 32, poor = 1, undifferentiated = 1	Not applicable	0.044	0.328
	Tumour budding	Present: 4/15 vs. 23/41	0.28 (0.08–1.04)	0.051	0.328
Family history suggestive of CRC predisposition †	Perineural/vascular invasion	Suggestive history: no invasion = 8, perineural only = 0, vascular only = 3, both = 2; negative history: no invasion = 9, perineural only = 8, vascular only = 9, both = 17	Not applicable	0.023	0.328
	Tumour budding	Present: 3/13 vs. 24/43	0.24 (0.06–0.99)	0.038	0.328
	T stage at diagnosis	Suggestive history: T1 = 3, T2 = 3, T3 = 8, T4 = 1, TX = 0; negative history: T1 = 4, T2 = 1, T3 = 30, T4 = 10, TX = 3	Not applicable	0.048	0.328

OR, odds ratio; CI, confidence interval; BH, Benjamini–Hochberg; CRC, colorectal cancer; MSI, microsatellite instability. The three comparisons with the lowest raw *p*-values are shown for each prespecified clinical feature. For binary outcomes, values represent patients with versus without the clinical feature. For multicategorical outcomes, the complete category distributions are shown, and the reported *p*-value refers to the corresponding global 2 × K comparison; therefore, no single OR is applicable. † Patients with unknown family-history information were excluded from the analysis. BH-adjusted *p*-values characterise the exploratory statistical evidence.

**Table 11:** Variance (PCA) and inertia (MCA).

Method	Component/Dimension	Eigenvalue	Variance/inertia, %
PCA	PC1	1.405	23.4
PCA	PC2	1.163	19.4
PCA	PC3	1.090	18.2
PCA	PC4	0.997	16.6
PCA	PC5	0.805	13.4
MCA, raw inertia	Dim.1	0.234	23.4
MCA, raw inertia	Dim.2	0.194	19.4
MCA, raw inertia	Dim.3	0.182	18.2
MCA, raw inertia	Dim.4	0.166	16.6
MCA, raw inertia	Dim.5	0.134	13.4
MCA, Benzécri-adjusted	Dim.1	0.007	82.6
MCA, Benzécri-adjusted	Dim.2	0.001	13.4
MCA, Benzécri-adjusted	Dim.3	<0.001	4.1

PCA, principal component analysis; MCA, Multiple correspondence analysis. Raw MCA inertia is presented for five dimensions and reproduces the variance proportions obtained by PCA. Benzécri-adjusted inertia is shown only for dimensions with positive adjusted eigenvalues. Adjusted MCA inertia should not be compared numerically with PCA variance or raw MCA inertia.

**Table 12:** Operational molecular pathway definitions.

Molecular Pathway	Genes Included in Both Assays †	Oncomine Precision GX5 Only	MiSeq-Based Assay Only
RAS/RAF/MAPK signalling	*BRAF*, *HRAS*, *KRAS*, *MAP2K1*, *MAP2K2*, *NRAS*, *RAF1*	—	—
Receptor tyrosine kinase signalling	*ALK*, *EGFR*, *ERBB2*, *ERBB3*, *ERBB4*, *FGFR2*, *FGFR3*, *KIT*, *MET*, *NTRK1*	*RET*	—
PI3K/AKT/mTOR signalling	*AKT1*, *PIK3CA*, *PTEN*	MTOR	—
WNT/β-catenin signalling	CTNNB1	—	—
TGF-β/SMAD signalling	No common coverage	—	*SMAD4*
TP53/cell-cycle regulation	*TP53*	*CDKN2A*, *CHEK2*	*FBXW7*
DNA damage repair/polymerase proofreading	*POLE*	*CHEK2*	—
G-protein signalling	*GNA11*, *GNAQ*	*GNAS*	—
Hedgehog signalling	No common coverage	SMO	—
Nuclear receptor signalling	ESR1	AR	—
Other non-canonical/exploratory alterations	*IDH1*	KEAP1	—

For descriptive pathway-level summaries, a pathway was considered affected when at least one P/LP alteration was detected in any covered gene assigned to that pathway. Genes not covered by the assay used were treated as untested. † For pooled inferential analyses, pathway status was defined exclusively using pathogenic or likely pathogenic alterations in genes shared by both NGS assays.

**Table 13:** Operational definition of clinical features used for subgroup analyses.

Clinical Feature	Operational Definition
Early diagnosis	Colorectal cancer diagnosed at ≤50 years
Significant polyposis	≥10 colorectal polyps documented before first colorectal surgery referral or during baseline diagnostic work-up
Previous malignant neoplasm	Any malignant neoplasm diagnosed before index colorectal cancer
Family history of colorectal cancer	Colorectal cancer documented in at least one relative of any degree
Family history suggestive of colorectal cancer predisposition	Any of the following: colorectal cancer in a relative diagnosed ≤50 years; two or more relatives with colorectal cancer; colorectal cancer across two or more generations; colorectal cancer plus a Lynch syndrome-associated tumour in the family; or explicit reference to Lynch syndrome, familial adenomatous polyposis, polyposis syndrome, hereditary colorectal cancer syndrome, Amsterdam criteria or Bethesda criteria in the clinical record
Usual presentation	Absence of all documented clinical features listed above

## Data Availability

The data presented in this study are not publicly available due to ethical and privacy restrictions imposed by the Institutional Ethics Committee of ULSSA and ICBAS. The datasets contain sensitive patient information and cannot be shared, in accordance with institutional policies and applicable data protection regulations.

## Supplementary Materials

The following supporting information can be downloaded at: https://www.mdpi.com/article/10.3390/ijms27156930/s1.

## Abbreviations

The following abbreviations are used in this manuscript:

AJCC	American Joint Committee on Cancer
*AKT1*	AKT serine/threonine kinase 1
*ALK*	Anaplastic lymphoma kinase
*APC*	Adenomatous polyposis coli
AR	Androgen receptor
BH	Benjamini–Hochberg
*BRAF*	B-Raf proto-oncogene, serine/threonine kinase
*CDKN2A*	Cyclin-dependent kinase inhibitor 2A
*CHEK2*	Checkpoint kinase 2
CI	Confidence interval
CIMP	CpG island methylator phenotype
CNS	Central nervous system
CNV	Copy-number variation
CRC	Colorectal cancer
CTNNB1	Catenin beta 1
CTx	Chemotherapy
DNA	Deoxyribonucleic acid
*EGFR*	Epidermal growth factor receptor
*ERBB2*	Erb-b2 receptor tyrosine kinase 2
*ERBB3*	Erb-b2 receptor tyrosine kinase 3
*ERBB4*	Erb-b2 receptor tyrosine kinase 4
ESMO	European Society for Medical Oncology
ESR1	Estrogen receptor 1
FDR	False discovery rate
*FBXW7*	F-box and WD repeat domain containing 7
*FGFR2*	Fibroblast growth factor receptor 2
*FGFR3*	Fibroblast growth factor receptor 3
*GNA11*	G protein subunit alpha 11
*GNAQ*	G protein subunit alpha q
*GNAS*	*GNAS* complex locus
HER2	Human epidermal growth factor receptor 2
*HRAS*	HRas proto-oncogene, GTPase
HRD	Homologous recombination deficiency
ICBAS	Instituto de Ciências Biomédicas Abel Salazar
*IDH1*	Isocitrate dehydrogenase 1
IQR	Interquartile range
KEAP1	Kelch-like ECH-associated protein 1
*KIT*	*KIT* proto-oncogene, receptor tyrosine kinase
*KRAS*	*KRAS* proto-oncogene, GTPase
*MAP2K1*	Mitogen-activated protein kinase kinase 1
*MAP2K2*	Mitogen-activated protein kinase kinase 2
*MET*	*MET* proto-oncogene, receptor tyrosine kinase
*MLH1*	MutL homolog 1
MMR	Mismatch repair
MSI	Microsatellite instability
MTOR	Mechanistic target of rapamycin kinase
NGS	Next-generation sequencing
*NRAS*	*NRAS* proto-oncogene, GTPase
*NTRK1*	Neurotrophic receptor tyrosine kinase 1
OPA	Oncomine Precision Assay
OR	Odds ratio
P/LP	Pathogenic/Likely Pathogenic
PC	Principal component
PCA	Principal component analysis
PCR	Polymerase chain reaction
*PIK3CA*	Phosphatidylinositol-4,5-bisphosphate 3-kinase catalytic subunit alpha
*POLE*	DNA polymerase epsilon, catalytic subunit
*PTEN*	Phosphatase and tensin homolog
*RAF1*	Raf-1 proto-oncogene, serine/threonine kinase
*RET*	Ret proto-oncogene
RT	Radiotherapy
SD	Standard deviation
*SMAD4*	SMAD family member 4
SMO	Smoothened, frizzled class receptor
TGF-β	Transforming growth factor beta
TMB	Tumour mutational burden
TNM	Tumour, node, metastasis
*TP53*	Tumour protein p53
ULSSA	Unidade Local de Saúde de Santo António
UMI	Unique molecular identifier
VAF	Variant allele frequency
WNT	Wnt signalling pathway
