# Supplementary material for: Next-Generation Sequencing in Colorectal Cancer: Real-World Molecular Profiling and Clinical Correlations
Source: Int J Mol Sci. 2026 Aug 1;27(15):6930. doi: 10.3390/ijms27156930 (PMC13466781; doi:10.3390/ijms27156930)
Supplement: Supplementary file 1 [file ijms-27-06930-s001.zip › ijms-4411928-supplementary.pdf]

Supplementary Materials

Next-Generation Sequencing in Colorectal Cancer: Real-World Molecular Profiling and Clinical Correlations

Tables

- **Table S1.** Gene-level NGS findings for all reportable alterations, with platform-specific valid denominators
- **Table S2.** Full catalogue of pathogenic and likely pathogenic sequence-level alterations
- **Table S3.** Full catalogue of variants of uncertain significance and unclassified sequence-level alterations
- **Table S4.** Silent variants
- **Table S5.** Copy-number variation events
- **Table S6.** Analytical presentation categories and variant classification of all reportable alteration events
- **Table S7.** Family-history sensitivity analyses with Unknown grouped with No: top three results in each analytical domain
- **Table S8.** Summary of the final Benjamini–Hochberg analytical families
- **Table S9.** Direct correspondence between PCA and MCA individual-score axes
- **Table S10.** Technical characteristics of the NGS assays used in the cohort
- **Table S11.** Specimen-level characteristics of the NGS cohort

Figures

- **Figure S1. MCA eigenvalue spectrum: raw versus Benzécri-corrected inertia**
- **Figure S2. MCA of P/LP common-gene profiles**

Table S1. Gene-level NGS findings for all reportable alterations, with platform-specific valid denominators.

Gene	Valid N	No alteration detected	Alteration detected	Missing	Gene	Valid N	No alteration detected	Alteration detected	Missing
TP53	97	43/97 (44.3%)	54/97 (55.7%)	0	AKT1	97	96/97 (99.0%)	1/97 (1.0%)	0
KRAS	97	57/97 (58.8%)	40/97 (41.2%)	0	CDKN2A	87	86/87 (98.9%)	1/87 (1.1%)	10
PIK3CA	97	78/97 (80.4%)	19/97 (19.6%)	0	CTNNB1	97	96/97 (99.0%)	1/97 (1.0%)	0
BRAF	97	86/97 (88.7%)	11/97 (11.3%)	0	EGFR	97	96/97 (99.0%)	1/97 (1.0%)	0
ERBB2	97	92/97 (94.8%)	5/97 (5.2%)	0	ESR1	97	96/97 (99.0%)	1/97 (1.0%)	0
ERBB4	97	92/97 (94.8%)	5/97 (5.2%)	0	FGFR3	97	96/97 (99.0%)	1/97 (1.0%)	0
NRAS	97	93/97 (95.9%)	4/97 (4.1%)	0	GNA11	97	96/97 (99.0%)	1/97 (1.0%)	0
PTEN	97	93/97 (95.9%)	4/97 (4.1%)	0	HRAS	97	96/97 (99.0%)	1/97 (1.0%)	0
ALK	97	94/97 (96.9%)	3/97 (3.1%)	0	IDH1	97	96/97 (99.0%)	1/97 (1.0%)	0
FBXW7	10	7/10 (70.0%)	3/10 (30.0%)	87	KEAP1	87	86/87 (98.9%)	1/87 (1.1%)	10
AR	87	85/87 (97.7%)	2/87 (2.3%)	10	MAP2K1	97	96/97 (99.0%)	1/97 (1.0%)	0
CHEK2	87	85/87 (97.7%)	2/87 (2.3%)	10	MAP2K2	97	96/97 (99.0%)	1/97 (1.0%)	0
ERBB3	97	95/97 (97.9%)	2/97 (2.1%)	0	MET	97	96/97 (99.0%)	1/97 (1.0%)	0
FGFR2	97	95/97 (97.9%)	2/97 (2.1%)	0	MTOR	87	86/87 (98.9%)	1/87 (1.1%)	10
GNAQ	97	95/97 (97.9%)	2/97 (2.1%)	0	NTRK1	97	96/97 (99.0%)	1/97 (1.0%)	0
GNAS	87	85/87 (97.7%)	2/87 (2.3%)	10	POLE	97	96/97 (99.0%)	1/97 (1.0%)	0
KIT	97	95/97 (97.9%)	2/97 (2.1%)	0	RET	87	86/87 (98.9%)	1/87 (1.1%)	10
RAF1	97	95/97 (97.9%)	2/97 (2.1%)	0	SMAD4	10	9/10 (90.0%)	1/10 (10.0%)	87
SMO	87	85/87 (97.7%)	2/87 (2.3%)	10					

Values are shown as n/N (%) using the number of valid cases for each gene. Missing values reflect genes without available data because of platform coverage. "No alteration detected" refers only to the analysed regions.

Table S2. Full catalogue of pathogenic and likely pathogenic sequence-level alterations.

Clinical significance	Gene	HGVSc	HGVSp	Events, n	Patients, n/N tested (%)	% of events within gene	Median VAF, %
Pathogenic	BRAF	c.1799T>A	p.V600E	8	8/97 (8.2%)	72.7%	20.5
Pathogenic	BRAF	c.1781A>G	p.D594G	1	1/97 (1.0%)	9.1%	7.7
Pathogenic	BRAF	c.1406G>C	p.G469A	1	1/97 (1.0%)	9.1%	33.9
Pathogenic	BRAF	c.1801A>G	p.K601E	1	1/97 (1.0%)	9.1%	36.0
Pathogenic	EGFR	c.2239_2248del	p.L747_A750de-linsP	1	1/97 (1.0%)	100.0%	32.6
Pathogenic	ERBB2	c.2264T>C	p.L755S	1	1/97 (1.0%)	50.0%	13.9
Pathogenic	ERBB2	c.2033G>A	p.R678Q	1	1/97 (1.0%)	50.0%	42.3
Pathogenic	GNAS	c.601C>T	p.R201C	1	1/87 (1.1%)	50.0%	35.4
Pathogenic	IDH1	c.394C>T	p.R132C	1	1/97 (1.0%)	100.0%	34.0
Pathogenic	KRAS	c.35G>A	p.G12D	18	18/97 (18.6%)	45.0%	25.9
Pathogenic	KRAS	c.35G>T	p.G12V	5	5/97 (5.2%)	12.5%	17.2
Pathogenic	KRAS	c.38G>A	p.G13D	6	6/97 (6.2%)	15.0%	31.9
Pathogenic	KRAS	c.436G>A	p.A146T	3	3/97 (3.1%)	7.5%	35.4
Pathogenic	KRAS	c.35G>C	p.G12A	2	2/97 (2.1%)	5.0%	28.0
Pathogenic	KRAS	c.34G>T	p.G12C	2	2/97 (2.1%)	5.0%	36.5
Pathogenic	KRAS	c.34G>A	p.G12S	1	1/97 (1.0%)	2.5%	20.9
Pathogenic	KRAS	c.351A>T	p.K117N	1	1/97 (1.0%)	2.5%	27.2
Pathogenic	KRAS	c.183A>C	p.Q61H	1	1/97 (1.0%)	2.5%	5.3
Pathogenic	NRAS	c.35G>T	p.G12V	1	1/97 (1.0%)	25.0%	40.0
Pathogenic	NRAS	c.183A>T	p.Q61H	1	1/97 (1.0%)	25.0%	22.2
Pathogenic	NRAS	c.181C>A	p.Q61K	1	1/97 (1.0%)	25.0%	19.0
Pathogenic	NRAS	c.182A>G	p.Q61R	1	1/97 (1.0%)	25.0%	15.8
Pathogenic	PIK3CA	c.1633G>A	p.E545K	3	3/97 (3.1%)	15.0%	24.1
Pathogenic	PIK3CA	c.3140A>G	p.H1047R	3	3/97 (3.1%)	15.0%	26.1
Pathogenic	PIK3CA	c.1624G>A	p.E542K	3	3/97 (3.1%)	15.0%	6.8
Pathogenic	PIK3CA	c.1636C>A	p.Q546K	2	2/97 (2.1%)	10.0%	16.8
Pathogenic	PIK3CA	c.115G>A	p.E39K	1	1/97 (1.0%)	5.0%	11.0
Pathogenic	PIK3CA	c.1625A>T	p.E542V	1	1/97 (1.0%)	5.0%	6.6
Pathogenic	PIK3CA	c.3140A>T	p.H1047L	1	1/97 (1.0%)	5.0%	15.1
Pathogenic	PIK3CA	c.3139C>T	p.H1047Y	1	1/97 (1.0%)	5.0%	15.7
Pathogenic	PIK3CA	c.320A>C	p.N107T	1	1/97 (1.0%)	5.0%	26.8
Pathogenic	PIK3CA	c.1035T>A	p.N345K	1	1/97 (1.0%)	5.0%	37.8
Pathogenic	PIK3CA	c.323G>A	p.R108H	1	1/97 (1.0%)	5.0%	18.5
Pathogenic	PIK3CA	c.113G>A	p.R38H	1	1/97 (1.0%)	5.0%	23.0
Pathogenic	POLE	c.1231G>C	p.V411L	1	1/97 (1.0%)	100.0%	17.6
Pathogenic	PTEN	c.469_470insG	p.E157GfsTer23	1	1/97 (1.0%)	25.0%	16.9
Pathogenic	PTEN	c.389G>A	p.R130Q	1	1/97 (1.0%)	25.0%	11.5
Pathogenic	RAF1	c.770C>T	p.S257L	1	1/97 (1.0%)	50.0%	10.7
Pathogenic	SMAD4	c.1081C>T	p.R361C	1	1/10 (10.0%)	100.0%	33.0
Pathogenic	TP53	c.733G>A	p.G245S	4	4/97 (4.1%)	6.7%	19.9

Clinical significance	Gene	HGVSc	HGVSp	Events, n	Patients, n/N tested (%)	% of events within gene	Median VAF, %
Pathogenic	TP53	c.637C>T	p.R213*	4	4/97 (4.1%)	6.7%	33.8
Pathogenic	TP53	c.817C>T	p.R273C	4	4/97 (4.1%)	6.7%	33.5
Pathogenic	TP53	c.818G>A	p.R273H	4	4/97 (4.1%)	6.7%	39.6
Pathogenic	TP53	c.844C>T	p.R282W	3	3/97 (3.1%)	5.0%	34.5
Pathogenic	TP53	c.742C>T	p.R248W	2	2/97 (2.1%)	3.3%	32.0
Pathogenic	TP53	c.404G>T	p.C135F	1	1/97 (1.0%)	1.7%	35.1
Pathogenic	TP53	c.404G>A	p.C135Y	1	1/97 (1.0%)	1.7%	34.6
Pathogenic	TP53	c.527G>T	p.C176F	1	1/97 (1.0%)	1.7%	33.8
Pathogenic	TP53	c.526T>C	p.C176R	1	1/97 (1.0%)	1.7%	7.5
Pathogenic	TP53	c.731G>A	p.G244D	1	1/97 (1.0%)	1.7%	37.9
Pathogenic	TP53	c.734G>A	p.G245D	1	1/97 (1.0%)	1.7%	43.9
Pathogenic	TP53	c.785G>T	p.G262V	1	1/97 (1.0%)	1.7%	46.2
Pathogenic	TP53	c.502del	p.H168TfsTer2	1	1/97 (1.0%)	1.7%	16.9
Pathogenic	TP53	c.578A>G	p.H193R	1	1/97 (1.0%)	1.7%	15.1
Pathogenic	TP53	c.716A>G	p.N239S	1	1/97 (1.0%)	1.7%	44.8
Pathogenic	TP53	c.455C>T	p.P152L	1	1/97 (1.0%)	1.7%	16.3
Pathogenic	TP53	c.529_546del	p.P177_C182del	1	1/97 (1.0%)	1.7%	67.8
Pathogenic	TP53	c.569C>T	p.P190L	1	1/97 (1.0%)	1.7%	29.4
Pathogenic	TP53	c.743G>T	p.R248L	1	1/97 (1.0%)	1.7%	30.5
Pathogenic	TP53	c.799C>T	p.R267W	1	1/97 (1.0%)	1.7%	53.8
Pathogenic	TP53	c.817C>A	p.R273S	1	1/97 (1.0%)	1.7%	42.8
Pathogenic	TP53	c.845G>A	p.R282Q	1	1/97 (1.0%)	1.7%	72.7
Pathogenic	TP53	c.916C>T	p.R306*	1	1/97 (1.0%)	1.7%	22.0
Pathogenic	TP53	c.428T>C	p.V143A	1	1/97 (1.0%)	1.7%	32.1
Pathogenic	TP53	c.469G>A	p.V157I	1	1/97 (1.0%)	1.7%	68.0
Pathogenic	TP53	c.437G>A	p.W146*	1	1/97 (1.0%)	1.7%	28.0
Pathogenic	TP53	c.659A>G	p.Y220C	1	1/97 (1.0%)	1.7%	67.0
Pathogenic	TP53	c.708C>A	p.Y236*	1	1/97 (1.0%)	1.7%	35.7
Pathogenic	TP53	c.560-1G>A	—	1	1/97 (1.0%)	1.7%	67.2
Likely pathogenic	AKT1	c.49G>A	p.E17K	1	1/97 (1.0%)	100.0%	54.6
Likely pathogenic	AR	c.2440T>G	p.F814V	1	1/87 (1.1%)	100.0%	43.9
Likely pathogenic	CHEK2	c.1107_1108insT	p.G370WfsTer25	1	1/87 (1.1%)	100.0%	15.0
Likely pathogenic	CTNNB1	c.133_135del	p.S45del	1	1/97 (1.0%)	100.0%	21.8
Likely pathogenic	ERBB3	c.310G>A	p.V104M	2	2/97 (2.1%)	100.0%	14.2
Likely pathogenic	FBXW7	c.1268G>T	p.G423V	1	1/10 (10.0%)	50.0%	19.0
Likely pathogenic	FBXW7	c.1394G>A	p.R465H	1	1/10 (10.0%)	50.0%	19.0
Likely pathogenic	GNAQ	c.623_624insG	p.Q209PfsTer13	1	1/97 (1.0%)	100.0%	4.5
Likely pathogenic	GNAS	c.681G>T	p.Q227H	1	1/87 (1.1%)	50.0%	4.6
Likely pathogenic	HRAS	c.178G>A	p.G60S	1	1/97 (1.0%)	100.0%	6.7
Likely pathogenic	KRAS	c.437C>T	p.A146V	1	1/97 (1.0%)	2.5%	25.5
Likely pathogenic	PIK3CA	c.3127A>G	p.M1043V	1	1/97 (1.0%)	5.3%	20.4

Clinical significance	Gene	HGVSc	HGVSp	Events, n	Patients, n/N tested (%)	% of events within gene	Median VAF, %
Likely pathogenic	PTEN	c.1022T>G	p.F341C	1	1/97 (1.0%)	25.0%	12.8
Likely pathogenic	PTEN	c.1026+2T>A	—	1	1/97 (1.0%)	25.0%	25.1
Likely pathogenic	RAF1	c.775T>C	p.S259P	1	1/97 (1.0%)	50.0%	6.6
Pathogenic / likely pathogenic	TP53	c.524G>A	p.R175H	6	6/97 (6.2%)	10.0%	36.0
Pathogenic / likely pathogenic	TP53	c.586C>T	p.R196*	2	2/97 (2.1%)	3.3%	55.3
Likely pathogenic	TP53	c.713del	p.C238LfsTer9	1	1/97 (1.0%)	1.7%	24.3
Likely pathogenic	TP53	c.725G>T	p.C242F	1	1/97 (1.0%)	1.7%	37.8
Likely pathogenic	TP53	c.672G>T	p.E224D	1	1/97 (1.0%)	1.7%	7.4
Likely pathogenic	TP53	c.581T>G	p.L194R	1	1/97 (1.0%)	1.7%	17.0
Likely pathogenic	TP53	c.741C>G	p.N247K	1	1/97 (1.0%)	1.7%	48.3
Likely pathogenic	TP53	c.747G>T	p.R249S	1	1/97 (1.0%)	1.7%	22.3
Likely pathogenic	TP53	c.800G>A	p.R267Q	1	1/97 (1.0%)	1.7%	10.5

Alterations are ordered by clinical significance and then by gene. Recurrent identical alterations are aggregated. Percent within gene uses the number of P/LP alteration events in that gene as denominator. The patient percentage uses the number of patients in whom the gene was tested. Silent variants and copy-number variations are excluded and tabulated separately.

Table S3. Full catalogue of variants of uncertain significance sequence-level alterations.

Gene	HGVSc	HGVSp	Events, n	Patients, n/N tested (%)	% of events within gene	Median VAF, %
ALK	c.1274G>T	p.C425F	1	1/97 (1.0%)	50.0%	7.3
ALK	c.4579C>T	p.P1527S	1	1/97 (1.0%)	50.0%	7.7
CDKN2A	c.160A>C	p.M54L	1	1/87 (1.1%)	100.0%	43.7
ERBB2	c.1157C>A	p.A386D	1	1/97 (1.0%)	33.3%	39.0
ERBB2	c.3182T>C	p.L1061P	1	1/97 (1.0%)	33.3%	49.0
ERBB2	c.1466C>T	p.P489L	1	1/97 (1.0%)	33.3%	39.0
ERBB4	c.884-8_884-7del	—	4	4/97 (4.1%)	57.1%	17.5
ERBB4	c.884-9_884-7del	—	2	2/97 (2.1%)	28.6%	17.5
ERBB4	c.884-10_884-7del	—	1	1/97 (1.0%)	14.3%	12.0
ESR1	c.898C>T	p.R300C	1	1/97 (1.0%)	100.0%	34.0
FBXW7	c.1309G>A	p.G437R	1	1/10 (10.0%)	100.0%	23.0
FGFR2	c.1168T>C	p.C390R	1	1/97 (1.0%)	100.0%	26.0
GNA11	c.541C>T	p.R181W	1	1/97 (1.0%)	100.0%	24.6
KIT	c.1615A>C	p.I539L	1	1/97 (1.0%)	50.0%	53.9
KIT	c.1495A>G	p.K499E	1	1/97 (1.0%)	50.0%	12.6
MAP2K1	c.700A>G	p.R234G	1	1/97 (1.0%)	100.0%	5.8
NTRK1	c.1739C>A	p.T580N	1	1/97 (1.0%)	100.0%	45.9
CHEK2	c.1141A>G	p.M381V	1	1/87 (1.1%)	100.0%	73.0
FGFR3	c.1195C>T	p.R399C	1	1/97 (1.0%)	100.0%	8.6
GNAQ	c.624_625del	p.Q209PfsTer22	1	1/97 (1.0%)	100.0%	4.4
KEAP1	c.1156G>A	p.G386S	1	1/87 (1.1%)	100.0%	42.4
MAP2K2	c.373T>A	p.C125S	1	1/97 (1.0%)	100.0%	3.6
MET	c.3685A>T	p.M1229L	1	1/97 (1.0%)	100.0%	21.1
MTOR	c.6016G>C	p.V2006L	1	1/87 (1.1%)	100.0%	39.3
PIK3CA	c.249T>G	p.F83L	1	1/97 (1.0%)	100.0 %	25.2
SMO	c.1417G>A	p.D473N	1	1/87 (1.1%)	100.0%	4.4

VUS and unclassified sequence-level alterations are presented separately from the principal P/LP analysis. Percent within gene uses the number of VUS/unclassified events in that gene as denominator. Silent variants are excluded and tabulated separately.

Table S4. Silent variants.

Gene	HGVSc	HGVSp	Events, n	Patients, n/N tested (%)	% of events within gene	Median VAF, %
ALK	c.1891T>C	p.L631L	1	1/97 (1.0%)	100.0%	5.6
ERBB2	c.2541C>T	p.A847A	1	1/97 (1.0%)	100.0%	57.0
FGFR2	c.759T>G	p.P253P	1	1/97 (1.0%)	100.0%	51.0
KRAS	c.*2483C>T	—	1	1/97 (1.0%)	100.0%	6.4
RET †	—	p.L769=;R770	1	1/87 (1.1%)	100.0%	11.4

Silent variants are listed independently of their clinical-significance annotation and were not included in the principal P/LP molecular analyses. † HGVSp notation reproduced verbatim from the original NGS report.

Table S5. Copy-number variation events.

Gene	Events, n	Patients, n/N tested (%)	% of events within gene
AR	1	1/87 (1.1%)	100.0%
PIK3CA	1	1/87 (1.1%)	100.0%
PTEN	1	1/87 (1.1%)	100.0%
SMO	1	1/87 (1.1%)	100.0%

Copy-number variations were assessed only by the Oncomine platform and only for selected genes; therefore, n = 87 is used as the denominator for the observed CNV genes. CNV genes were not included in sequence-level P/LP, VUS, silent-variant, VAF, or lollipop analyses.

Table S6. Analytical presentation categories and variant classification of all reportable alteration events.

A. Analytical presentation category

Category	Alteration events, n	% of all 198 events
P/LP sequence-level alteration	159	80.3%
VUS	30	15.2%
Silent variant	5	2.5%
Copy-number variation	4	2.0%
Total	198	100.0%

B. Variant classification

Category	Alteration events, n	% of all 198 events
Missense mutation	162	81.8%
Frameshift deletion	10	5.1%
Nonsense mutation	9	4.5%
Silent variant	5	2.5%
Copy-number variation	4	2.0%
Frameshift insertion	3	1.5%
In-frame deletion	2	1.0%
Splice-site variant	2	1.0%
In-frame insertion	1	0.5%

Panel A reproduces inside the table the mutually exclusive analytical categories used to construct Supplementary Tables S2–S5. Panel B classifies the same 198 events by variant type. P/LP, pathogenic or likely pathogenic; VUS, variant of uncertain significance.

Table S7. Family-history sensitivity analyses with Unknown grouped with No: top three results in each analytical domain.

Sensitivity analysis	Domain	Outcome	Comparison, n/N vs n/N	OR (95% CI)	Raw p	Adjusted p	Test
Family history of CRC; Unknown grouped with No	Pathway	RAS/RAF/MAPK	6/17 vs 47/80	0.38 (0.13–1.14)	0.078	1.000	Pearson χ^2
Family history of CRC; Unknown grouped with No	Pathway	PI3K/AKT/mTOR	3/17 vs 19/80	0.69 (0.18–2.65)	0.755	1.000	Fisher exact
Family history of CRC; Unknown grouped with No	Pathway	TP53/cell-cycle/checkpoint	10/17 vs 44/80	1.17 (0.40–3.38)	0.773	1.000	Pearson χ^2
Family history of CRC; Unknown grouped with No	Gene	CHEK2	1/14 vs 0/73	16.33 (0.63–422.45)	0.161	1.000	Fisher exact
Family history of CRC; Unknown grouped with No	Gene	BRAF	0/17 vs 11/80	0.17 (0.01–3.07)	0.203	1.000	Fisher exact
Family history of CRC; Unknown grouped with No	Gene	ERBB2	1/17 vs 1/80	4.94 (0.29–83.12)	0.321	1.000	Fisher exact
Family history of CRC; Unknown grouped with No	Clinicopathological	Tumour budding	4/15 vs 39/71	0.30 (0.09–1.03)	0.047	0.326	Pearson χ^2
Family history of CRC; Unknown grouped with No	Clinicopathological	Perineural/vascular invasion (global)	Global 2×K comparison; valid n=87	Not applicable	0.059	0.326	Pearson χ^2 (global, df=3)
Family history of CRC; Unknown grouped with No	Clinicopathological	Tumour differentiation (global)	Global 2×K comparison; valid n=82	Not applicable	0.072	0.326	Pearson χ^2 (global, df=3)
Suggestive family history; Unknown grouped with No	Pathway	RAS/RAF/MAPK	6/15 vs 47/82	0.50 (0.16–1.52)	0.215	1.000	Pearson χ^2
Suggestive family history; Unknown grouped with No	Pathway	Receptor tyrosine kinase signalling	1/15 vs 4/82	1.39 (0.14–13.40)	0.577	1.000	Fisher exact
Suggestive family history; Unknown grouped with No	Pathway	TP53/cell-cycle/checkpoint	8/15 vs 46/82	0.89 (0.30–2.70)	0.843	1.000	Pearson χ^2
Suggestive family history; Unknown grouped with No	Gene	CHEK2	1/12 vs 0/75	19.70 (0.76–513.19)	0.138	1.000	Fisher exact
Suggestive family history; Unknown grouped with No	Gene	BRAF	0/15 vs 11/82	0.20 (0.01–3.59)	0.205	1.000	Fisher exact
Suggestive family history; Unknown grouped with No	Gene	ERBB2	1/15 vs 1/82	5.79 (0.34–97.97)	0.287	1.000	Fisher exact
Suggestive family history; Unknown grouped with No	Clinicopathological	Tumour budding	3/13 vs 40/73	0.25 (0.06–0.97)	0.035	0.326	Pearson χ^2
Suggestive family history; Unknown grouped with No	Clinicopathological	Perineural/vascular invasion (global)	Global 2×K comparison; valid n=87	Not applicable	0.102	0.367	Pearson χ^2 (global, df=3)
Suggestive family history; Unknown grouped with No	Clinicopathological	Tumour differentiation (global)	Global 2×K comparison; valid n=82	Not applicable	0.154	0.401	Pearson χ^2 (global, df=3)

These analyses are separate from the primary family-history analyses, which exclude Unknown. For each family-history definition and domain, the three lowest raw p-values are displayed. Multicategorical Pearson 2×K results are exploratory where contingency tables are sparse. No result remained significant after BH.

Table S8. Summary of the final Benjamini–Hochberg analytical families.

Analytical family	Tests, n	Minimum raw p	Minimum adjusted p	Raw p < 0.05, n	Adjusted p < 0.05, n	Interpretation
Clinicopathological	45	0.009	0.328	6	0	No result remained significant after BH.
Molecular P/LP genes	103	0.041	1.000	1	0	No result remained significant after BH.
Pathways P/LP	38	0.108	1.000	0	0	No result remained significant after BH.
Clinicopathological FH Sensitivity	18	0.035	0.326	2	0	No result remained significant after BH.
Mol. P/LP genes FH Sensitivity	42	0.138	1.000	0	0	No result remained significant after BH.
Mol. Pathways P/LP FH Sensitivity	16	0.078	1.000	0	0	No result remained significant after BH.

Across the six prespecified analytical families, nine comparisons had raw $p < 0.05$, but none remained significant after BH correction. The minimum adjusted p-value was 0.326.

Table S9. Direct correspondence between PCA and MCA individual-score axes.

PCA axis	MCA axis	Score correlation, r	Absolute r	PCA/MCA scale ratio	Interpretation
PC1	Dim.1	−1.000	1.000	−√6	Same axis; sign reversed
PC2	Dim.2	1.000	1.000	√6	Same axis
PC3	Dim.3	1.000	1.000	√6	Same axis
PC4	Dim.4	−1.000	1.000	−√6	Same axis; sign reversed
PC5	Dim.5	1.000	1.000	√6	Same axis
PC6	Dim.6	1.000	1.000	√6	Same axis

Correlations were calculated between patient-level FactoMineR PCA and MCA scores. An absolute correlation of 1.000 on every matched axis, with a constant scale factor of √6 and arbitrary sign reversals, shows that PCA and MCA recover the same patient configuration for this complete six-gene binary matrix. MCA therefore acts as a categorical sensitivity representation rather than an independent contradictory result. Raw MCA inertia proportions match PCA variance proportions; Benzécri-adjusted inertia is a separate correction and should not be compared numerically with PCA variance.

Table S10. Technical characteristics of the NGS assays used in the cohort.

Feature	Oncomine™ Precision GX5 Solid Tumour DNA and Fusions	MiSeq-based assay
Platform	Ion Torrent Genexus System	MiSeq, Illumina
Specimen type	Formalin-fixed paraffin-embedded tumour tissue or diagnostic biopsy material	Formalin-fixed paraffin-embedded tumour tissue or diagnostic biopsy material
Panel used	Oncomine™ Precision GX5 Solid Tumour DNA and Fusions	QIAseq Solid Tumour Panel, custom QIAGEN panel
Alteration classes assessed	DNA hotspot variants, selected copy-number variations, selected intergenic fusions/rearrangements, and selected intragenic fusions/rearrangements	Somatic single-nucleotide variants, insertions, deletions
DNA hotspot genes / genes tested	AKT1, AKT2, AKT3, ALK, AR, ARAF, BRAF, CDK4, CDKN2A, CHEK2, CTNNB1, EGFR, ERBB2, ERBB3, ERBB4, ESR1, FGFR1, FGFR2, FGFR3, FGFR4, FLT3, GNA11, GNAQ, GNAS, HRAS, IDH1, IDH2, KEAP1, KIT, KRAS, MAP2K1, MAP2K2, MET, MTOR, NRAS, NTRK1, NTRK2, NTRK3, PDGFRA, PIK3CA, POLE, PTEN, RAF1, RET, ROS1, SMO and TP53	AKT1, ALK, BRAF, CTNNB1, DDR2, EGFR, ERBB2, ERBB3, ERBB4, ESR1, FBXW7, FGFR1, FGFR2, FGFR3, FLT3, GNA11, GNAQ, HRAS, IDH1, IDH2, KIT, KRAS, MAP2K1, MAP2K2, MET, MYD88, NOTCH1, NRAS, NTRK1, PDGFRA, PIK3CA, POLE, PTEN, RAF1, ROS1, SMAD4, STK11 and TP53
CNV genes	ALK, AR, CD274, CDKN2A, EGFR, ERBB2, ERBB3, FGFR1, FGFR2, FGFR3, KRAS, MET, PIK3CA, PTEN and SMO.	Not tested
Fusion/rearrangement genes	Intergenic fusions involving ALK, BRAF, ESR1, FGFR1, FGFR2, FGFR3, MET, NRG1, NTRK1, NTRK2, NTRK3, NUTM1, RET, ROS1, RSPO2 and RSPO3; intragenic fusions involving AR, EGFR and MET	Not assessed
Analytical sensitivity / reporting threshold	5% variant allele frequency	5% variant allele frequency; variants with allele frequency below 5% are not reported
Quality / coverage criteria	Morphological control with sufficient neoplastic cell representation in the selected material; analytical sensitivity reported as 5%	Mean UMI read Q score ≥38 and UMI coverage of regions of interest ≥100× in at least 90% of reads
Bioinformatic analysis / interpretation	Variant reporting according to local molecular pathology workflow	Secondary analysis performed using QIAGEN CLC Genomics Workbench; variant interpretation using QCI Interpret; variants reported according to HGVS nomenclature and classified according to ACMG recommendations
Main technical limitations stated in report	Absence of a reportable alteration should be interpreted only within the analysed genomic regions and assay limits	Does not exclude pseudogene or highly homologous sequence interference, low-coverage regions, mutations outside analysed regions, or variants not detectable by the method used; sensitivity may be lower in regions with UMI coverage below recommended thresholds

Table S11. Specimen-level characteristics of the NGS cohort (n = 97).

**Characteristic**	**n/N (%)**
**Specimen source**	
Surgical resection specimen	82/97 (84.5%)
Pre-operative diagnostic biopsy (neoadjuvant setting) **a**	5/97 (5.2%)
Pre-operative diagnostic biopsy (non-neoadjuvant) **b**	8/97 (8.2%)
Metastatic lesion biopsy **c**	2/97 (2.1%)
**Prior treatment exposure at time of sampling**	
None (treatment-naïve)	96/97 (99.0%)
Prior systemic therapy d	1/97 (1.0%)

a Patients with rectal cancer who underwent diagnostic biopsy prior to initiation of neoadjuvant chemoradiotherapy; NGS was performed on the pre-treatment biopsy specimen.
b Includes patients managed without surgical resection (palliative intent, n = 3), pre-operative endoscopic biopsy (n = 1), and biopsy-confirmed diagnosis without neoadjuvant therapy (n = 4).
c One abdominal peritoneal metastasis and one hepatic metastasis, both biopsy-confirmed.
d One patient underwent NGS on a hepatic metastasis biopsy obtained after multiple prior lines of systemic therapy. All remaining 96 patients were treatment-naïve at the time of specimen collection.
NGS, next-generation sequencing.

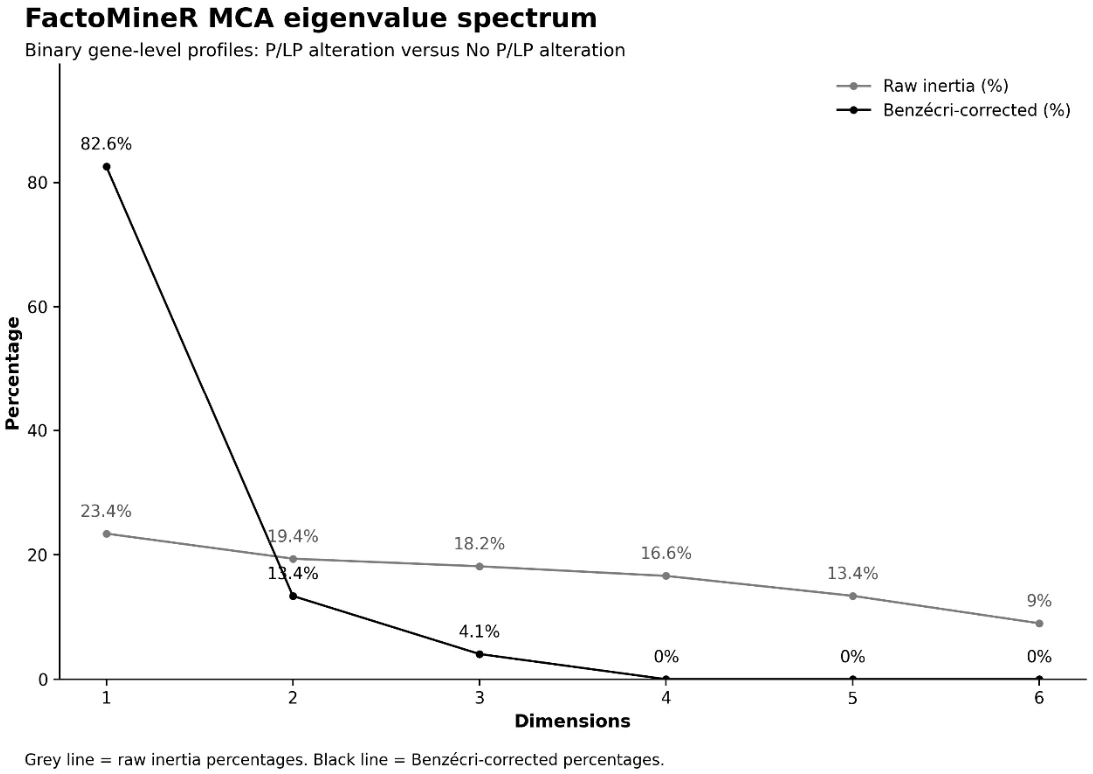

Figure S1. MCA eigenvalue spectrum: Raw vs. Benzécri-corrected inertia.

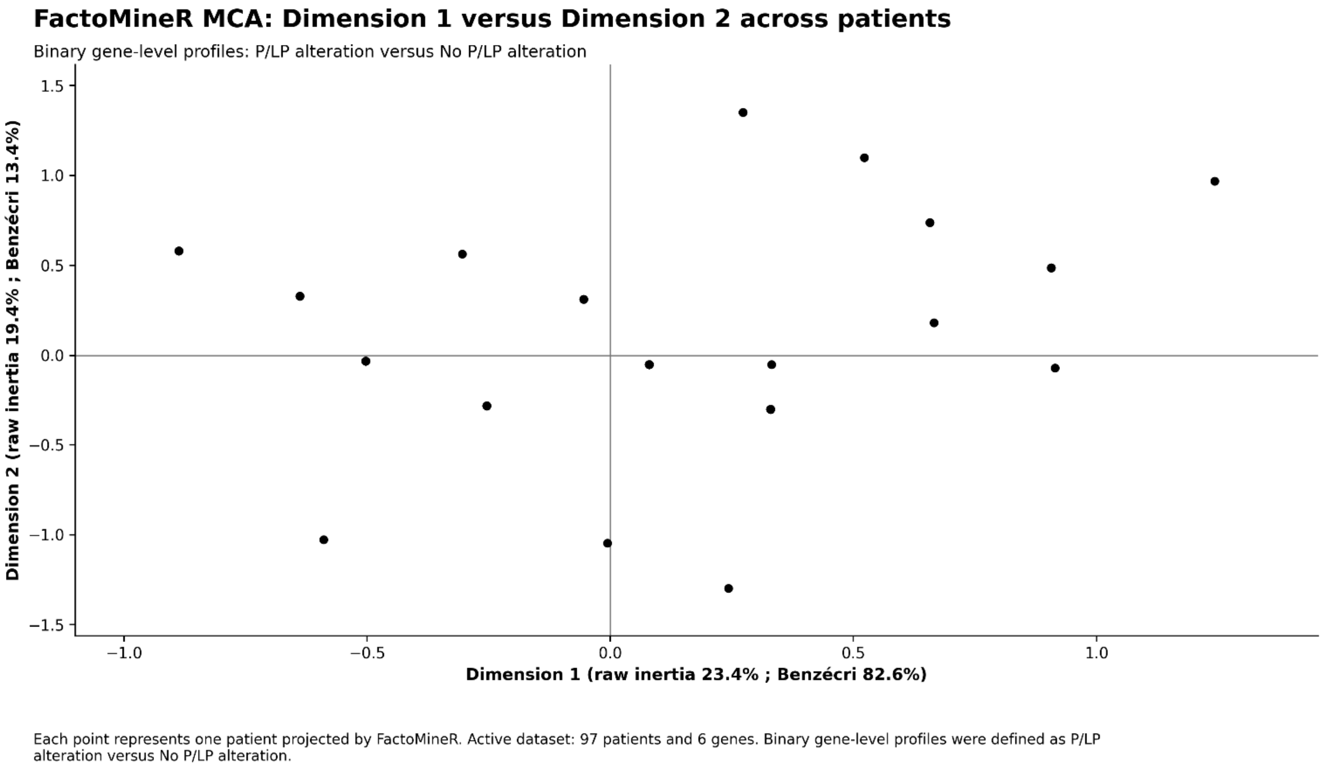

Figure S2. MCA of P/LP common-gene profiles.
